# Evolutionary Dynamics Based on Comparative Genomics of Pathogenic Escherichia coli Lineages Harboring Polyketide Synthase (*pks*) Island

**DOI:** 10.1128/mBio.03634-20

**Published:** 2021-03-02

**Authors:** Arya Suresh, Sabiha Shaik, Ramani Baddam, Amit Ranjan, Shamsul Qumar, Savita Jadhav, Torsten Semmler, Irfan A. Ghazi, Lothar H. Wieler, Niyaz Ahmed

**Affiliations:** a Pathogen Biology Laboratory, Department of Biotechnology and Bioinformatics, University of Hyderabad, Hyderabad, India; b Department of Microbiology, Dr. D. Y. Patil Medical College, Hospital and Research Centre (Dr. D. Y. Patil Vidyapeeth), Pune, India; c Robert Koch Institute, Berlin, Germany; d Department of Plant Sciences, University of Hyderabad, Hyderabad, India; Louis Stokes Veterans Affairs Medical Center

**Keywords:** colibactin, *pks* island, polyketide synthase, genotoxins, *Escherichia coli*, *Escherichia* toxins, genomics, pathogenicity islands, phylogeny

## Abstract

The genotoxin colibactin is a secondary metabolite produced by the polyketide synthase (*pks*) island harbored by extraintestinal pathogenic E. coli (ExPEC) and other members of the *Enterobacteriaceae* that has been increasingly reported to have critical implications in human health. The present study entails a high-throughput whole-genome comparison and phylogenetic analysis of such pathogenic E. coli isolates to gain insights into the patterns of distribution, horizontal transmission, and evolution of the island. For the current study, 23 *pks*-positive ExPEC genomes were newly sequenced, and their virulome and resistome profiles indicated a preponderance of virulence encoding genes and a reduced number of genes for antimicrobial resistance. In addition, 4,090 E. coli genomes from the public domain were also analyzed for large-scale screening for *pks*-positive genomes, out of which a total of 530 *pks-*positive genomes were studied to understand the subtype-based distribution pattern(s). The *pks* island showed a significant association with the B2 phylogroup (82.2%) and a high prevalence in sequence type 73 (ST73; *n* = 179) and ST95 (*n* = 110) and the O6:H1 (*n* = 110) serotype. Maximum-likelihood (ML) phylogeny of the core genome and intergenic regions (IGRs) of the ST95 model data set, which was selected because it had both *pks*-positive and *pks*-negative genomes, displayed clustering in relation to their carriage of the *pks* island. Prevalence patterns of genes encoding RM systems in the *pks*-positive and *pks-*negative genomes were also analyzed to determine their potential role in *pks* island acquisition and the maintenance capability of the genomes. Further, the maximum-likelihood phylogeny based on the core genome and *pks* island sequences from 247 genomes with an intact *pks* island demonstrated horizontal gene transfer of the island across sequence types and serotypes, with few exceptions. This study vitally contributes to understanding of the lineages and subtypes that have a higher propensity to harbor the *pks* island-encoded genotoxin with possible clinical implications.

## INTRODUCTION

Pathogenic Escherichia coli strains possess many different virulence factors in varied repertoires involved in subverting host cell mechanisms to enable persistence in otherwise protected environments of the host, with the capability to develop severe forms of pathogenesis that lead to high morbidity and mortality (1, 2). Mobile genetic element-enabled horizontal gene transfer (HGT), inactivation of antivirulence genes (3), and point mutation-derived functional alterations significantly contribute to the evolution of virulence in E. coli (2). Genes encoding different virulence factors, such as toxins, adhesins, iron acquisition systems, and capsules, could possibly be carried on or shuttled through mobile genetic elements, genomic islands, phages, and plasmids. These genes are capable of undergoing the horizontal gene transfer occurring among compatible organisms (4, 5) and could be abundantly distributed in extraintestinal pathogenic E. coli (ExPEC) strains. Genomic islands composed of large genomic regions (>10 kb), often flanked by repeat structures and carrying cryptic or functional mobility factors (integrases, transposases, etc.), display association with tRNA genes and possess distinct G+C contents (6). A subset of genomic islands called pathogenicity islands (PAIs) confer “quantum leaps” in the evolution of bacterial virulence by carrying numerous virulence-associated factors and enable adaptive evolution through horizontal gene transfer (7, 8). Colibactin is one such PAI-encoded genotoxic, nonribosomal peptide-polyketide secondary metabolite observed in uropathogenic, commensal, and neonatal meningitis-causing strains of E. coli (9). This metabolite was observed to induce double-stranded DNA breaks in eukaryotic cells, causing cell cycle arrest at the G_2_-M phase and chromosomal aberrations (10, 11) and contributing to severe clinical manifestations like meningitis (12) and sepsis (9, 13).

Colibactin biosynthesis is carried out by an assembly line machinery located in the *pks* genomic island (54 kb) which consists of 19 genes comprising of nonribosomal peptide megasynthases (NRPS; *clbH*, *clbJ*, and *clbN*), polyketide megasynthases (PKS; *clbC*, *clbI*, and *clbO*), two hybrid NRPS-PKS (*clbB* and *clbK*), and nine accessory and tailoring enzymes (10). A recent study has described the regulatory role of *clbR*, a LuxR-type DNA-binding helix-turn-helix (HTH) domain as a key transcriptional activator involved in the expression of the colibactin biosynthetic gene cluster (14). The *pks* island, with an increased G+C content compared to the core genome, was reported to be integrated into the *asnW* tRNA locus and flanked by direct repeats of 16 bp together with P4-like bacteriophage integrase genes (10, 15). These integrative elements function to transfer genetic determinants to other members of *Enterobacteriaceae* (10, 15). The *pks* island is observed to be present in pathogenic, commensal, and even probiotic bacterial strains (16). It was also observed to be present in members of the Enterobacteriaceae other than E. coli, such as Citrobacter koseri, Klebsiella pneumoniae, and Klebsiella aerogenes (15). Colorectal cancer (CRC) biopsy samples were shown to display increased prevalence of the *pks* island-harboring E. coli (17, 18). E. coli isolates having *pks* islands were found in more than half of the patients with familial adenomatous polyps, and their colonic biofilms could enhance carcinogenesis through mucus degradation, followed by adherence and augmented colonization (19). In addition to their postulated role in CRC progression, numerous studies describe the *pks* islands as virulence factors with clinical implications entailing systemic infection, neonatal meningitis, and lymphopenia (12, 20–22).

So far, only a few studies have attempted to understand the pattern of transfer and evolution of the *pks* island and its coevolution with the genome that harbors it. Enterobacterial repetitive intergenic consensus (ERIC) and random amplified polymorphic DNA (RAPD)-based genetic fingerprinting of *pks*-positive E. coli isolates obtained from human intestinal polyps showed diverse clustering patterns that implied their potential ability to colonize different environments (23). Another study performed bioinformatics analyses that unraveled the high prevalence of the *pks* island among Escherichia species, with close similarity of the *pks* island of E. coli with those of K. aerogenes, K. pneumoniae, and C. koseri (24). The combination of *in silico* and *in vitro* studies performed on the Escherichia coli Reference (ECOR) collection demonstrated that the immobile PAI group, i.e., those devoid of any transfer or mobility regions, comprising of high-pathogenicity island (HPI), *pks*, and *serU*, undergoes horizontal gene transfer “*en bloc*” along with the neighboring chromosomal backbone; this was observed to be F′-mediated transfer (25). The high homology within *pks* island sequences also conveyed the recent acquisition of the *pks* island (25). We attempted to employ a large-scale pangenome and phylogenetic analysis to comprehensively study and contribute insights to the distribution and evolutionary dynamics of this pathogenic island of clinical significance. The prevalence of *pks* island among ExPEC isolates from India and their genetic and functional characterization have been previously described by our group (26). The present study aims at describing the genome-wide comparisons and phylogenetic analysis of the *pks* island-carrying E. coli isolates from a previously described in-house collection, as well as the genome data obtained from the public domain. The study describes the distribution of *pks* island-harboring E. coli among phylogroups, sequence types, and serogroups, followed by pangenome and phylogenetic analyses with particular reference to genomes belonging to sequence type 95 (ST95) to understand the evolution and acquisition of this island. Phylogenetic analyses have also been performed to study the fine structure of island evolution with respect to the core genome and to understand the pattern of transfer and acquisition of the island. A preliminary study on the potential role of the distribution pattern of restriction modification systems towards the successful HGT and maintenance of *pks* islands has also been performed. We have employed large scale, whole-genome-based investigations for understanding the pathogenic *pks* islands with respect to their patterns of prevalence or preponderance and evolution among E. coli populations.

## RESULTS

### Genome characteristics.

Whole-genome sequencing of 23 *pks-*positive E. coli isolates that were previously characterized (26) was performed in the current study. The genomes showed an approximate size of 5.1 Mb with an average G+C content of 50.4%. The average number of coding sequences (CDS) was ∼5,000, displaying a coding percentage of 87%. The 23 *pks-*positive genomes analyzed here for the first time revealed distribution among the following different sequence types: ST12 (*n* = 6), ST73 (*n* = 4), ST827 (*n* = 3), ST14 (*n* = 3), ST998 (*n* = 3), ST1057 (*n* = 2), ST83 (*n* = 1), and ST127 (*n* = 1). The assembly statistics and genome sequence characteristics are summarized in Tables S1 and S2 in the supplemental material. The GenBank accession numbers of the 23 newly sequenced genomes have also been listed in Table S2. Whole-genome comparison of the 23 in-house *pks-*positive genomes was performed using BLAST Ring Image Generator (BRIG) (27) with the complete genome of strain IHE3034 as the reference (see Fig. S1a in the supplemental material). Results from the BRIG analysis indicated that the genomes shared a high degree of similarity, and variable regions were mostly identified as phages (denoted as black arcs). The *pks* island (denoted as a red arc) was also found to be conserved throughout the genomes. The island sequences reconstructed from the respective genomes were used as the query, along with the *pks* island sequence from IHE3034 as the reference in BRIG (27) (Fig. S1b). The island sequence was also annotated, and the individual genes of the island are depicted in the outermost ring (Fig. S1b). It was observed that the island sequences showed a high degree of conservation among the genomes, with variations only in the flanking regions in a few cases.

10.1128/mBio.03634-20.1 FIG S1 (a) Whole-genome comparative analysis of 23 *pks*-positive genomes using BRIG, with strain IHE3034 as the reference. Each ring represents a genome and the rings have been color coded based on the sequence type, and the genome names along with sequence types have been labeled. In addition, phages and the *pks* island have also been annotated in the outermost ring. (b)The *pks* island from 23 genomes reconstructed from the aligned reads was used as query against the *pks* island along with flanking regions from IHE3034 as the reference. Each ring represents an island from each *pks*-positive genome, and the rings have been color coded based on the sequence type of the genome. The genes of the island have also been annotated and are represented in the outermost ring. Download FIG S1, TIF file, 2.9 MB. Copyright © 2021 Suresh et al. 2021 Suresh et al. https://creativecommons.org/licenses/by/4.0/ This content is distributed under the terms of the Creative Commons Attribution 4.0 International license.

10.1128/mBio.03634-20.4 TABLE S1 Assembly and scaffolding statistics of the *pks*-positive genomes. Download Table S1, PDF file, 0.08 MB. Copyright © 2021 Suresh et al. 2021 Suresh et al. https://creativecommons.org/licenses/by/4.0/ This content is distributed under the terms of the Creative Commons Attribution 4.0 International license.

10.1128/mBio.03634-20.5 TABLE S2 Genome characteristics of *pks*-positive isolates. Download Table S2, PDF file, 0.1 MB. Copyright © 2021 Suresh et al. 2021 Suresh et al. https://creativecommons.org/licenses/by/4.0/ This content is distributed under the terms of the Creative Commons Attribution 4.0 International license.

### Virulome and resistome profiling of in-house *pks*-positive genomes.

**FIG 1:**
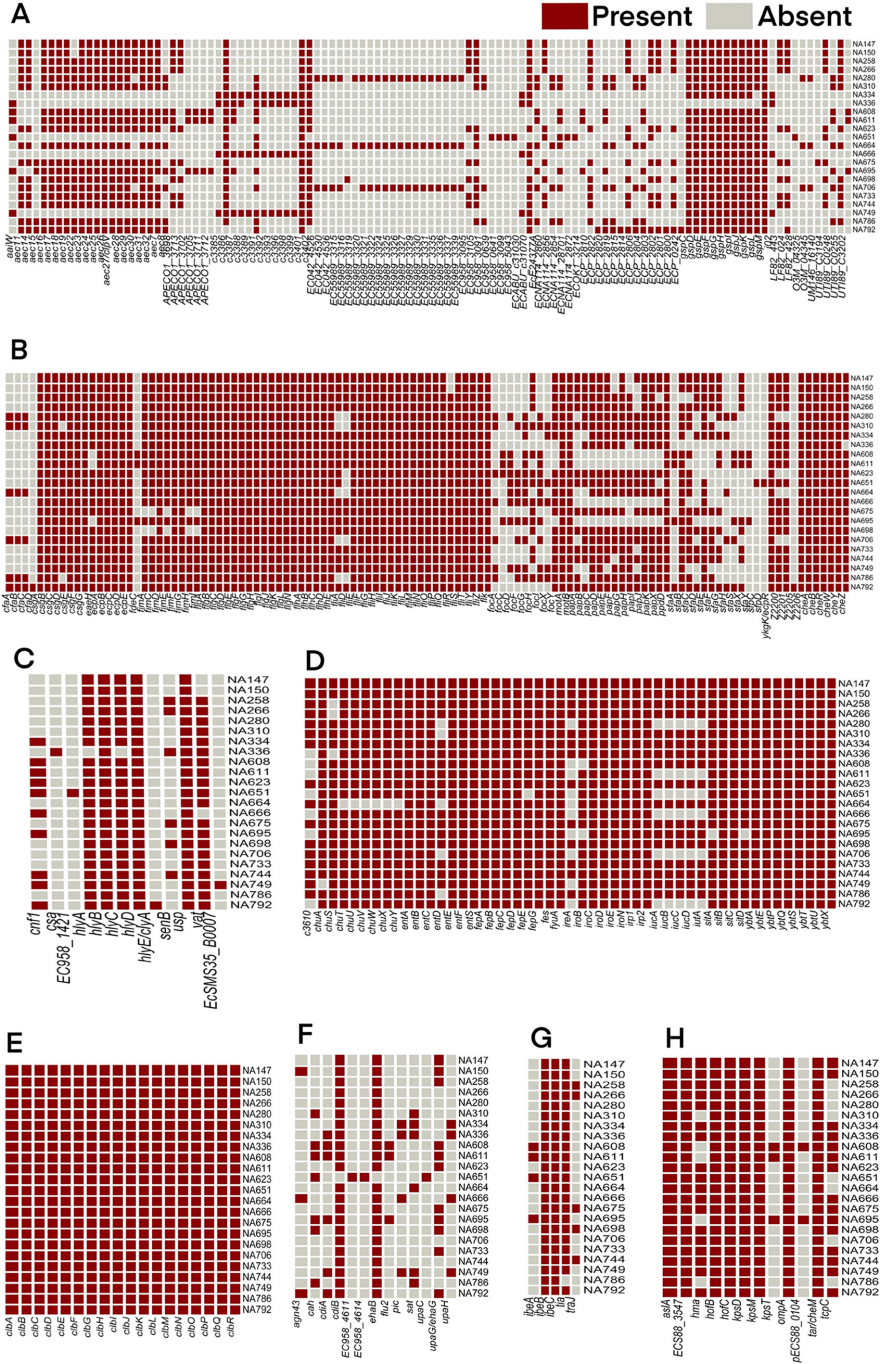
Heat map depicting the virulence profile of 23 in-house *pks-*positive isolates, depicting the presence and absence of 333 virulence genes belonging to different categories. (A) Secretory system, (B) adherence factors, (C) toxins, (D) siderophores/iron acquisition systems, (E) *pks* island genes, (F) autotransporters, G) invasins, and (H) others (genes corresponding to columns, from left to right, may be read in a sequential reading frame, such that each gene name aligns correctly with a single, corresponding column).

The in-house *pks-*positive genomes were screened to identify the prevalence of various virulence associated and antibiotic resistance conferring gene coordinates to determine the pathogenic potential of the corresponding ExPEC isolates. *In silico* virulence profiling using the Virulence Factor Database (VFDB) (28) showed these *pks-*positive isolates to have an abundance of adherence factors, type VI secretion systems, and siderophores, as depicted in the heat map (Fig. 1). Among the category of adherence genes, the *csgABCDEFG* gene complex involved in curli fiber production, assembly and transport, E. coli common pilus genes (*ecpABCDE*), and the type I fimbrial protein genes *fimABCDEFGHI* were found distributed in most of the genomes. In addition, peritrichous flagellar proteins (encoded by *flg*, *fli*, and *flh*), flagellar motor proteins (*motA* and *motB*), and chemotaxis proteins (*cheABRWYZ*) were also found to be present in the majority of the genomes. The invasin protein genes *ibeB*, *ibeC* and *tia* were found in most of the 23 isolates studied. Among secretion systems, genes coding for type VI secretion systems (98 out of the total of 111 genes belonging to the category of secretion systems) were observed in the greatest abundance, followed by genes encoding general secretory pathway proteins (*gspCDEFGHIJKLM*). Among the type VI secretion systems, *aec7, aec16, aec 17, aec 18, aec 19, aec 23, aec 24, aec 25, aec 26, aec 27, aec 28, aec 29, aec 30, aec 31, aec 32*, *c3386*, *c3401*, *c3402*, and *ECABU_c310170* were present in 18 or more genomes out of the 23 *pks*-positive genomes. Yersiniabactin siderophore system genes *ybtAEXPQRSTU*, *irp1*, and *irp2* were found to be present in all the isolates. Most of the genomes harbored other siderophore systems like *chuASTUVWXY*, enterobactin synthase genes *entABCDEFS*, ferrienterobactin transporter genes *fepABCDEG*, enterobactin esterase gene *fes*, and salmochelin genes *iroBCDEN.* It was also observed that 17/23 genomes harbored the aerobactin siderophore synthesis system genes, *iucABCD * and *iutA*. Among toxin genes, hemolysin-encoding genes *hlyABCD*, the uropathogenic-specific protein gene *usp*, and the hemoglobin protease gene *vat* were present in ∼23 genomes. In addition, the cyclomodulin cytotoxic necrotizing factor gene *cnf-1* was present in 10/23 isolates. Analysis using VFDB also confirmed the presence of *pks* island genes in all of the 23 genomes, indicating the integrity of the island in the genomes (Fig. 1). The comparison of the virulence profile of the in-house *pks-*positive genomes with that of the in-house *pks*-negative genomes has been described in Table S3 in the supplemental material.

10.1128/mBio.03634-20.6 TABLE S3 *In silico* virulence profile of the 23 *pks*-positive in-house genomes along with 23 *pks-*negative in-house genomes obtained using BLASTp analysis against the Virulence Factor Database (VFDB). In the dataset, 1 indicates presence and 0 indicates absence of the virulence factor in the corresponding genome. Download Table S3, XLSX file, 0.08 MB. Copyright © 2021 Suresh et al. 2021 Suresh et al. https://creativecommons.org/licenses/by/4.0/ This content is distributed under the terms of the Creative Commons Attribution 4.0 International license.

**FIG 2:**
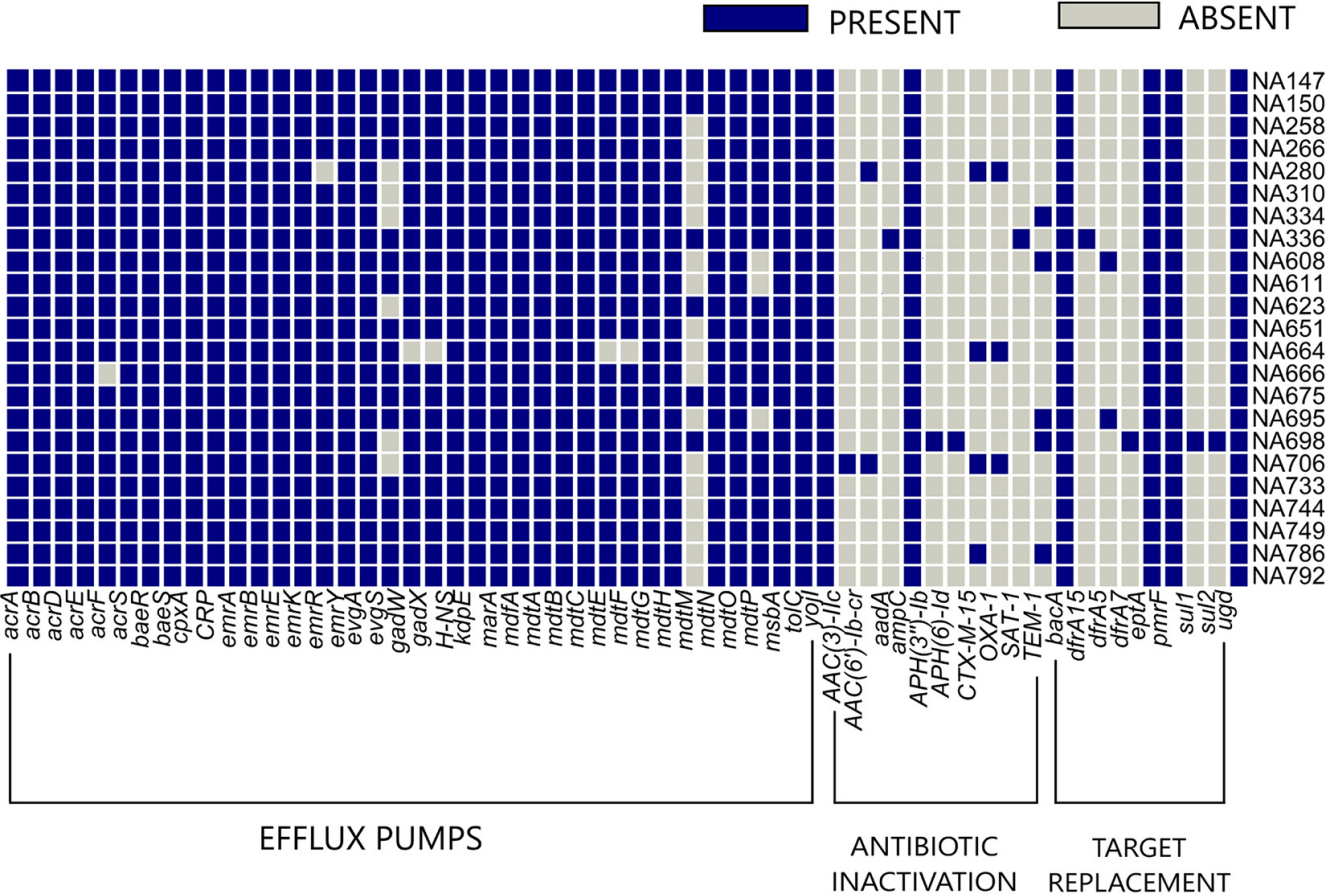
Heat map showing the presence and absence of 57 antibiotic resistance genes in 23 in-house *pks-*positive genomes. Blue and gray boxes indicate the presence and absence of the resistance gene, respectively. Gene names are represented on the *x* axis and isolate names on the *y* axis. The genes have also been categorized according to the mechanism of action against antimicrobials (gene names correspond to columns from left to right; they may be read in a sequential reading frame beginning *acrA* aligning to the left most column to *ugd* aligning with the right most column, in the heat map).

*In silico* antimicrobial gene profiling revealed that the majority of the resistance genes carried by *pks-*positive, in-house isolates belonged to the nonspecific antibiotic efflux pumps category (Fig. 2). The majority of the efflux pumps, including the aminoglycoside efflux pump (*acr*), two-component regulatory system (*baeSR*), global regulator (*CRP*), electrochemical gradient-powered transporter *emr*, and multiple antibiotic resistance family *mar*, were found to be prevalent in most of the genomes. The multidrug efflux system *mdt*, coupled with *gadX* and *gadW*, which offer resistance to penams, fluoroquinolones, and macrolides, were also observed in most *pks-*positive isolates. In the category of antibiotic inactivation, *ampC*, a class C beta-lactamase that encodes resistance against penicillins and cephalosporins, was also found to be present in all the isolates. Other beta-lactamases like CTX-M-15 (*n* = 4), OXA-1 (*n* = 3), and TEM-1 (*n* = 5) were detected in a few isolates. Antibiotic target replacement genes like the bacitracin resistance gene *bacA* and the coordinates from the gene family encoding phosphoethanolamine transferase (*ugd* or *pmrE*, *eptA* or *pmrC*, and *pmrF*) offering resistance against cationic antimicrobial peptides were found distributed in all the genomes (Fig. 2). The comparison of the resistance profile of the in-house *pks-*positive genomes with that of the in-house *pks*-negative genomes has been described in the Table S4 in the supplemental material.

10.1128/mBio.03634-20.7 TABLE S4 *In silico* resistance profile of the 23 *pks*-positive in-house genomes along with 23 *pks-*negative in-house genomes obtained using BLASTp analysis against the Comprehensive Antibiotic Resistance Database (CARD). In the dataset, 1 indicates presence and 0 indicates absence of the antibiotic resistance gene in the corresponding genome. Download Table S4, XLSX file, 0.02 MB. Copyright © 2021 Suresh et al. 2021 Suresh et al. https://creativecommons.org/licenses/by/4.0/ This content is distributed under the terms of the Creative Commons Attribution 4.0 International license.

### Prevalence and distribution of *pks-*positive E. coli.

A total of 4,113 genomes of E. coli were analyzed, of which 306 were complete and 3,753 were draft genomes downloaded from NCBI; 31 genomes were in-house or sequenced as a part of previous studies, whereas 23 genomes, as described, were the newly sequenced genomes taken for the present work. A total of 530 genomes were found positive for the presence of *pks* island genes and were designated with in-house identifiers (IDs) (*pks*p001 to *pks*p530) (the genome list, in-house IDs, and the accession numbers of these genomes obtained from NCBI and used for further analyses have been described in Tables S5 and S6 in the supplemental material). Among the 530 genomes, 247 genomes carried all 19 genes of the island, while 184 and 80 genomes carried 18 and 17 genes, respectively. The remaining 19 genomes carried fewer than 17 genes. Out of the 530 *pks-*positive genomes, the island (54 kb in size) was observed to be present in a single contig in 247 genomes. All of the *pks-*positive genomes carried 14 to 19 *pks* island genes, and the rest of the genomes did not harbor any of the *pks* island genes and hence were designated *pks*-negative.

10.1128/mBio.03634-20.8 TABLE S5 Accession numbers and isolate names of 530 *pks*-positive NCBI genomes used in the study. Genomes denoted in italics (*pksp001* to *pksp023*) represent the newly sequenced genomes for the study. Genomes belonging to ST95 (*n* = 110) are denoted with an asterisk (*) in their IDs. Download Table S5, PDF file, 0.3 MB. Copyright © 2021 Suresh et al. 2021 Suresh et al. https://creativecommons.org/licenses/by/4.0/ This content is distributed under the terms of the Creative Commons Attribution 4.0 International license.

10.1128/mBio.03634-20.9 TABLE S6 Isolate names and accession identifiers (IDs) of in-house *pks*-negative genomes using *in silico* virulence/resistance gene profiling (Sl. no. 1 to 23) and ST95 *pks*-negative genomes from NCBI (Sl. no. 24 to 72). Download Table S6, PDF file, 0.1 MB. Copyright © 2021 Suresh et al. 2021 Suresh et al. https://creativecommons.org/licenses/by/4.0/ This content is distributed under the terms of the Creative Commons Attribution 4.0 International license.

*In silico* multilocus sequence typing (MLST) revealed that there is a higher prevalence of the *pks* genomic island in sequence types ST73 (*n* = 179) and ST95 (*n* = 110), followed by ST127 (*n* = 52) and ST12 (*n* = 48) (Table 1). Interestingly, all ST73 isolates were observed to harbor the *pks* island, and none of the genomes belonging to the highly successful clonal group ST131 carried the island sequence. The percent prevalence of *pks-*positive genomes among sequence types ST95, ST127, and ST12 was 69.18% (110/159), 78.78% (52/66), and 97.9% (48/49), respectively. *In silico* phylogrouping revealed that the majority of the isolates (82%) belonged to the B2 phylogroup, indicating a strong association of the *pks* island-harboring E. coli isolates with the B2 phylogroup (Table 1), similar to the observations from PCR-based phylogrouping of the in-house isolates in our previous study, which showed that the majority of the isolates belonged to the B2 phylogroup (97%) (26).

**TABLE 1:** Sequence type, phylogroup, and serotype distribution of *pks-*positive genomes (*n* = 530) obtained from NCBI

Subtype	% (no.)
Phylogroup	
B2	81.69 (433)
A	0.56 (3)
Unknown	17.73 (94)
Sequence type	
ST73	33.8 (179)
ST95	20.7 (110)
ST127	9.8 (52)
ST12	9.1 (48)
ST141	3.6 (19)
ST998	3.02 (16)
ST404	2.07 (11)
ST80	1.7 (9)
Miscellaneous	12.6 (67)
Unknown	3.6 (19)
Serogroup	
O6:H1	20.7 (110)
O6:H31	9 (48)
O4:H5	8.6 (46)
O18:H7	7.5 (40)
O2:H6	6.8 (36)
O1:H7	6.4 (34)
O2:H1	6 (32)
O2:H7	6 (32)
O75:H5	4.3 (23)
O22:H1	3.7 (20)
O4:H1	3.7 (20)
O25:H1	3.2 (17)
O2:H4	3 (16)
O18:H1	2.2 (12)
Miscellaneous	7.5 (40)

*In silico* determination of serotypes using ECTyper displayed a higher prevalence of *pks*-positives in certain serotypes. The O6:H1 serotype was shown to have the highest number of *pks-*positive genomes (*n* = 110), followed by O6:H31 (*n* = 48), O4:H5 (*n* = 46), and O18:H7 (*n* = 40). The prevalence pattern of *pks-*positive genomes in different serotypes has been described in Table 1. Serotypes and sequence types with fewer than 10 genomes were grouped as “miscellaneous.”

### Pangenome analysis of ST95 genomes.

The ST95 group was observed to have both *pks*-positive and *pks-*negative genomes and thus was considered a suitable model data set in this study. Comparison between the *pks*-positives and *pks*-negatives from ST95 could help in providing insights into the potential acquisition and maintenance of *pks* island. A total of 3,057 genes constituted the core of 159 ST95 genomes, which included 110 *pks* positives and 49 *pks* negatives. These genes were subjected to clusters of orthologous groups (COG) classification using EggNOG (29), where 2,337 out of 3,057 genes were assigned to different COG classes, and the results are depicted in Table S7 in the supplemental material.

10.1128/mBio.03634-20.10 TABLE S7 Table showing the clusters of orthologous genes (COG) classification of core genes from 159 ST95 genomes. Download Table S7, PDF file, 0.1 MB. Copyright © 2021 Suresh et al. 2021 Suresh et al. https://creativecommons.org/licenses/by/4.0/ This content is distributed under the terms of the Creative Commons Attribution 4.0 International license.

### Core genome phylogeny of ST95.

**FIG 3:**
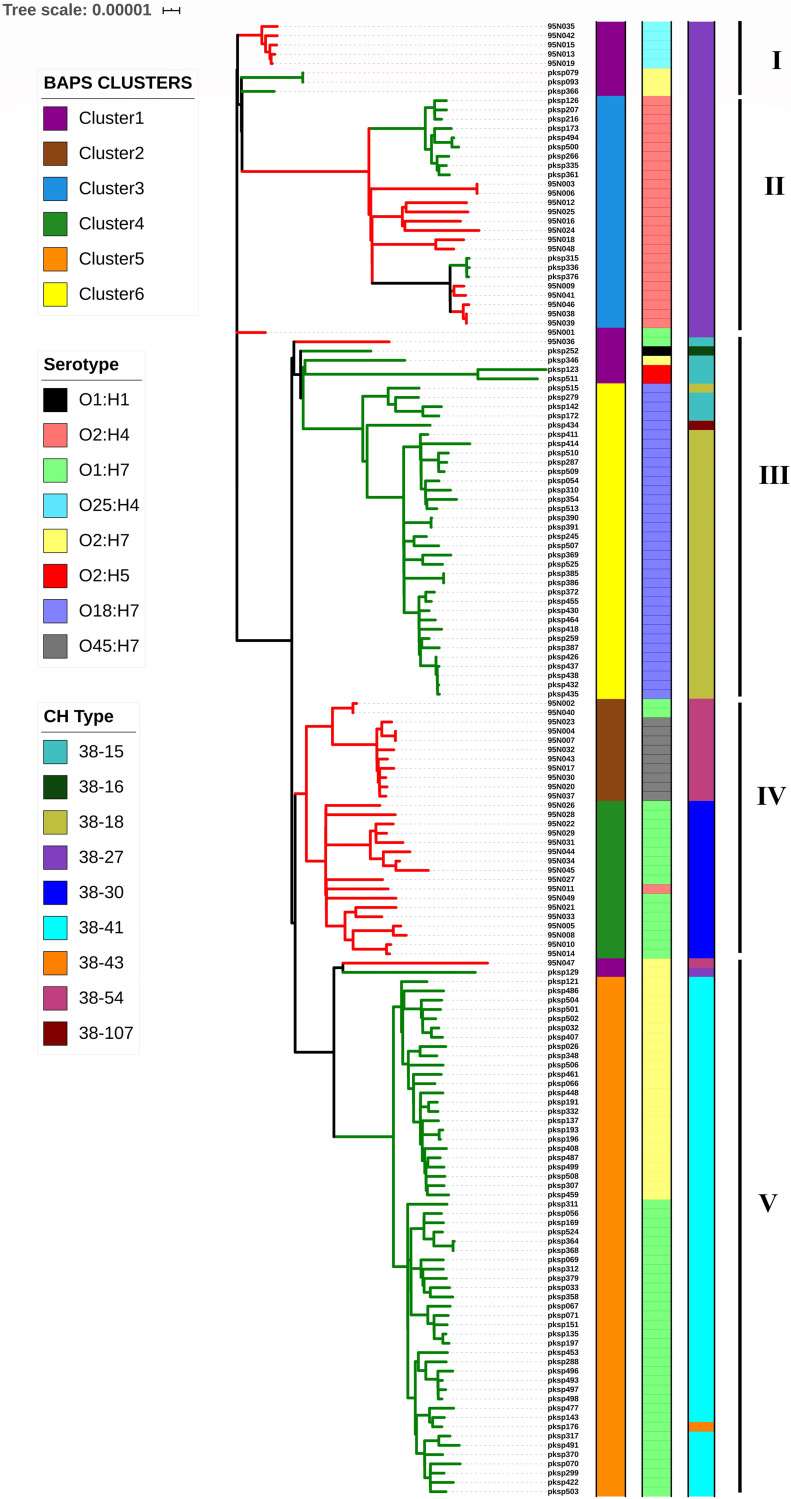
Maximum-likelihood core genome phylogeny of 159 ST95 isolates constructed using IQ-TREE and ClonalFrameML and visualized using iTOL. A total of five clades were observed (I to V). Green and red branches represent genomes positive and negative for the *pks* island, respectively. The first data strip represents the BAPS clusters, the second data strip represents the serotypes of the genomes as identified by ECTyper, and the third represents the CH types of the respective genomes.

Core genome maximum-likelihood (ML) phylogeny obtained from IQ-TREE (30) consisted of 5 different clades, where green branches denote *pks*-positives and red ones denote *pks*-negatives (Fig. 3). Clades I and II were observed to comprise both *pks-*positive and *pks*-negative genomes with mixed clading pattern(s). Clades III and V were found to consist predominantly of *pks-*positive genomes, except for one *pks-*negative genome each, whereas clade IV consisted of only *pks-*negative genomes. The distinct clustering of *pks-*positive and *pks*-negative isolates in a core genome-based phylogeny hinted towards the role of core genome in the acquisition and maintenance of the *pks* island (a part of accessory genome). All of the major clades of the ST95 core genome phylogenetic tree (Fig. 3) had bootstrap support values ranging from 89% to 100%. The core genome phylogeny of 159 ST95 genomes, along with an outgroup (ED1a), is depicted in Fig. S2 in the supplemental material.

10.1128/mBio.03634-20.2 FIG S2 Maximum-likelihood core genome phylogeny of 159 sequence type 95 (ST95) isolates, along with one outgroup genome, ED1a, constructed using IQ-TREE and ClonalFrameML and visualized using iTOL. Green and red branches represent genomes positive and negative for the *pks* island, respectively. The first data strip represents the serotypes of the genomes as identified by ECTyper, and the second represents the CH types of the genomes. Download FIG S2, TIF file, 1.3 MB. Copyright © 2021 Suresh et al. 2021 Suresh et al. https://creativecommons.org/licenses/by/4.0/ This content is distributed under the terms of the Creative Commons Attribution 4.0 International license.

The 159 isolates were grouped into six different clusters using hierBAPS (31) in the first level of clustering. The Bayesian analysis of population structure (BAPS) clusters were in concordance with maximum-likelihood phylogeny clades obtained from IQ-TREE (30) and were represented in the first data strip of Fig. 3. BAPS clusters 5 and 6 belonged to the *pks*-positive clades V and III, respectively, whereas BAPS clusters 2 and 4 belonged to the *pks-*negative clade IV. Genomes forming BAPS clusters 1 and 3 mostly belonged to clades I and II, which showed mixed clading of both *pks-*positive and *pks*-negative isolates (Fig. 3).

*In silico* serotyping using ECTyper classified the ST95 isolates into eight different serotypes. The branching pattern in the ML phylogeny was revealed to be mostly based on serovars of E. coli and also showed association with the island’s prevalence. The serotypes O18:H7 and O2:H7 comprised of mostly *pks-*positive isolates and the mixed clade belonged to O2:H4. Interestingly, O1:H7 isolates formed two separate clades and BAPS clusters, one of which was *pks*-positive and the other *pks*-negative (Fig. 3).

*In silico* CH typing (32) revealed that all of the ST95 genomes belonged to the same C type, 38, and variations were shown in the type I fimbrial gene *fimH*, which classified the 159 genomes into nine different CH types (Fig. 3). Clades I and II, which comprised the *pks-*positive and *pks*-negative mixed cluster belonged to CH type 38-27. *pks-*positive clade III (except 95N035) carried genomes belonging to CH types 38-18, 38-15, 38-16, and 38-107. The *pks-*negative clade IV comprised of genomes belonging to CH types 38-54 and 38-30. Clade V, which was predominantly *pks*-positive, belonged to CH type 38-41, except the genomes 95N044, *pks*p129 and *pks*p176, which belonged to CH types 38-54, 38-27, and 38-43, respectively (Fig. 3).

### Pangenome-wide analysis using Scoary for ST95 genomes.

A pangenome-wide analysis of accessory genes was performed using Scoary (33) to identify genes that could have a potential correlation to the *pks* island presence in the genome (Table 2). Genomes belonging to clade IV, which was an exclusively *pks*-negative clade, were compared to the rest of the genomes, which belonged to *pks-*positive and mixed clades. The genes that displayed differential prevalence and enrichment in the two sets of genomes, i.e., the ones which were completely absent in clade IV *pks*-negatives but were present in almost all the other genomes and vice versa are documented in Table 2, along with their prevalence details and functional annotations. Putative acetyltransferase gene *yjgM*, toxin-antitoxin biofilm protein gene *tabA_2*, and ornithine carbamoyltransferase chain I gene *argI_1* were each observed to be present as two different orthologs due to their sequence variation in each of these two groups of genomes analyzed. The prevalence of these genes across the *pks-*positive (*n* = 530) and *pks*-negative (*n* = 3,583) genomes were also evaluated, and the results are displayed in Table 2.

**TABLE 2:** Results of pangenome-wide analysis of ST95 genomes using Scoary^*a*^

Sl. no.	Gene	Non-unique gene name	Annotation	ST95 Scoary results (no. of genomes)		Prevalence analysis [no. (%)] among the entire dataset	
				Prevalence among *pks-*positives (*n* = 110) and *pks-*negative genomes (*n* = 20) from mixed clade	Prevalence among genomes from *pks-*negative clade (*n* = 29)	Prevalence among *pks* positives (*n* = 530)	Prevalence among *pks* negatives (*n* = 3,583)
1	*ybcF_2*		Putative carbamate kinase	130	0	476 (89.8)	261 (7.28)
2	*argI_1*		Ornithine carbamoyltransferase chain I	130	0	477 (90)	269 (7.50)
3	*tabA_2*		Toxin-antitoxin biofilm protein	130	0	477 (90)	264 (7.36)
4	*yjgM*		Putative acetyltransferase	130	0	522 (98.4)	261 (7.28)
5	*arcA*		Arginine deiminase	130	0	477 (90)	259 (7.2)
6	*argR_2*		ArgR-*arg*	129	0	476 (89.9)	264 (7.3)
7	*idnO*		5-Keto-d-gluconate 5-reductase	128	0	440 (83)	1,128 (31.4)
8	*idnD*		l-Idonate 5-dehydrogenase	128	0	440 (83)	1,129 (31.5)
9	*idnR*		IdnR transcriptional regulator	128	0	440 (83)	1,126 (31.4)
10	*idnK*		d-Gluconate kinase, thermosensitive	128	0	440 (83)	1,131 (31.56)
11	*idnT*		l-Idonate/5-ketogluconate/gluconate transporter IdnT	128	0	440 (83)	1,133 (31.62)
12	group_8070		Hypothetical protein	128	0	482 (90.9)	358 (9.9)
13	*yfcC_2*		Putative inner membrane protein; putative *S*-transferase	127	0	477 (90)	263 (7.34)
14	group_212	*tabA_2*	Toxin-antitoxin biofilm protein	0	29	39 (7.3)	3,298 (92.04)
15	*bdcA*		c-di-GMP binding protein involved in biofilm dispersal	0	29	39 (7.3)	3,244 (90.53)
16	group_1863		Hypothetical protein	0	28	41 (7.73)	3,153 (87.99)
17	*bdcR*		Putative transcriptional regulator	0	28	39 (7.3)	3,271 (91.29)
18	group_3605	*yjgM*	Putative acetyltransferase	0	28	50 (9.4)	3,563 (99.44)
19	group_7174	*hsdR*	Type 1 restriction enzyme R protein	0	28	122 (23)	484 (13.5)
20	group_803		Hypothetical protein	0	28	146 (27.5)	989 (27.6)
21	group_2253	*mdtM*	Multidrug efflux transporter	0	28	135 (25)	3,201 (89.3)
22	group_2075	*hsdM*	Host modification; DNA methylase M	0	27	122 (23)	484 (13.5)

a The analysis was performed between the *pks-*positive and mixed clades (clades I, II, III, and V) in comparison with the exclusively *pks*-negative clade (clade IV). The prevalence of the differentially enriched genes in the entire data set of *pks-*positive (*n* = 530) and *pks-*negative genomes (*n* = 3,583) is also shown.

### Intergenic region analysis of ST95 genomes.

**FIG 4:**
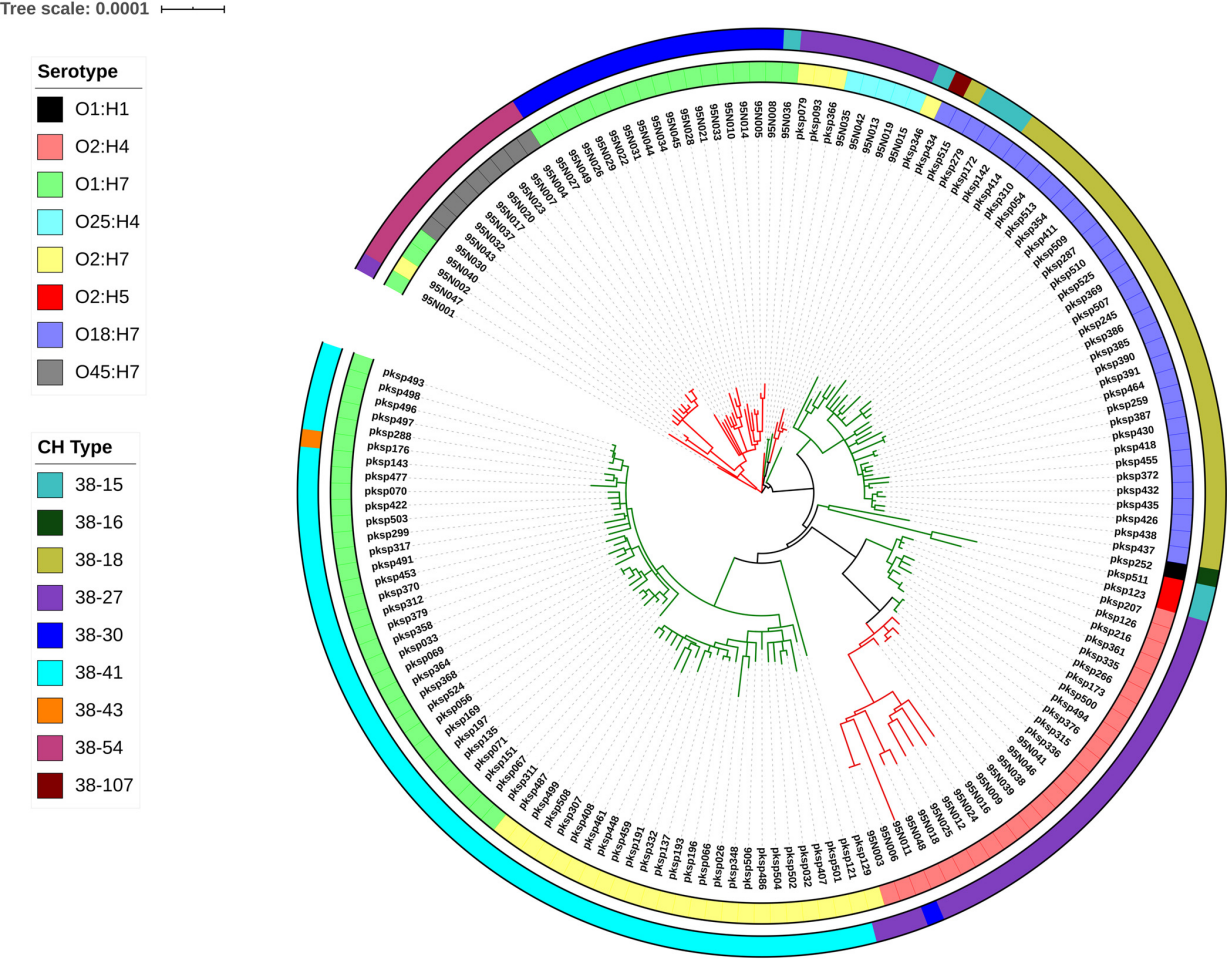
Maximum-likelihood intergenic region phylogeny of 159 ST95 isolates constructed using IQ-TREE and visualized using iTOL. Green and red branches represent *pks* island positives and negatives, respectively. The inner ring represents the serotypes of the genomes as identified by ECTyper, and the outer ring represents CH types of the genomes obtained from CHTyper.

Intergenic regions (IGRs), although they comprise of noncoding DNA sequences, form an important part of the bacterial genome with abundantly distributed regulatory regions which play a crucial role in the phenotypic variations in the bacteria (34). The analysis of core IGR regions in addition to the coding counterpart of the genome provides an improved resolution to the evolutionary analysis of bacteria. The analysis of core IGR phylogeny was performed to ascertain the correlation of sequence variation of the intergenic region to *pks* island distribution pattern(s). The core IGR phylogeny constructed using core IGR sequences extracted by Piggy (35) (Fig. 4) showed a clading pattern reflective of the carriage of the *pks* island more distinctly compared to the core genome phylogeny, with the *pks-*negative cluster found to clade separately from *pks-*positive and mixed clades. The IGR clades from O1:H7 *pks-*positive and *pks-*negative genomes were also found to be more distinct compared to the core genome phylogeny. All of the major clades of the ST95 IGR phylogenetic tree (Fig. 4) had bootstrap values ranging from 92.7% to 100%.

### RM system analysis.

**FIG 5:**
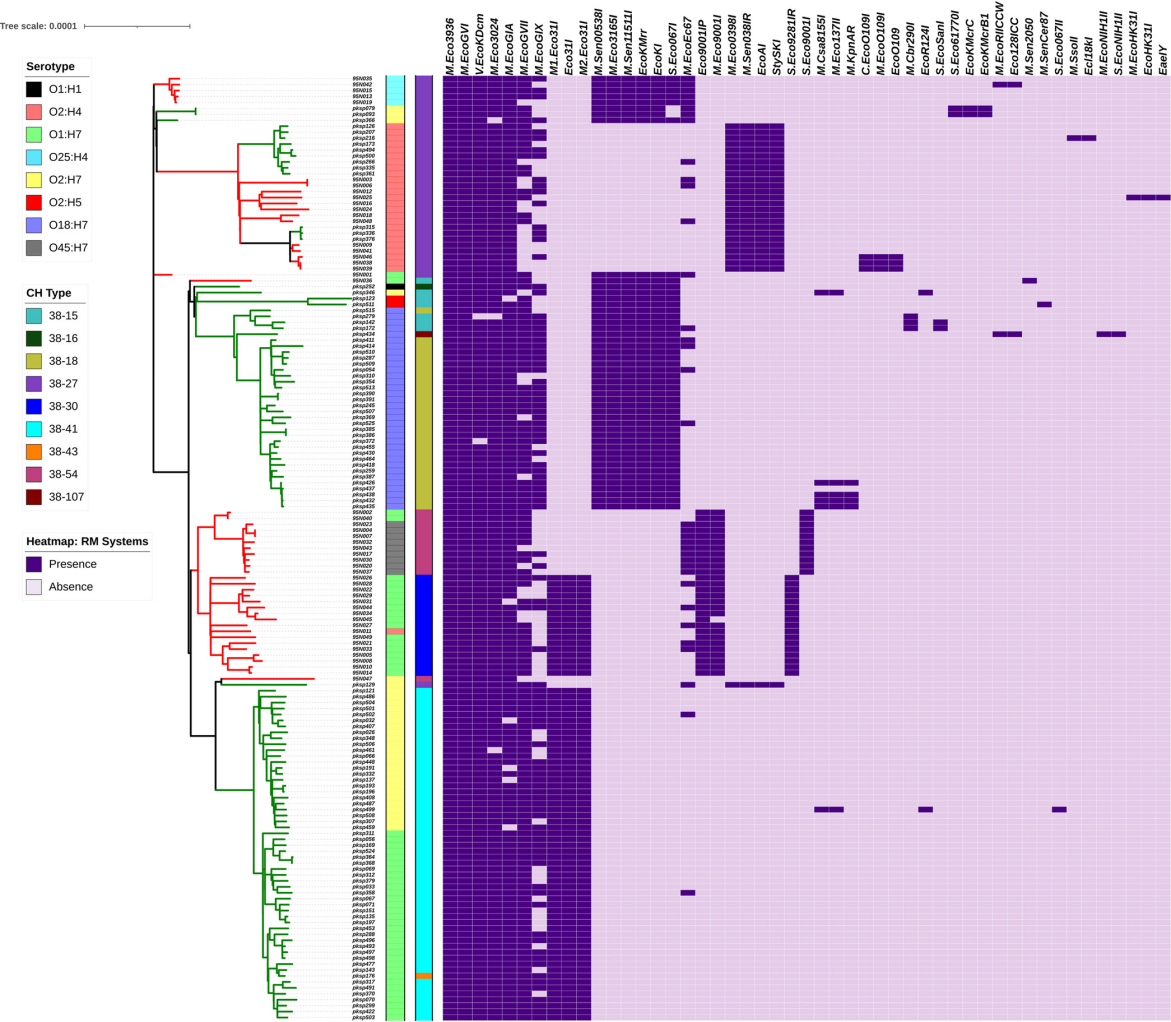
RM system prevalence pattern of ST95 genomes plotted as a heat map, along with core genome phylogeny with serotype and CH type labeled. The prevalence pattern showed accordance with the clading pattern of the phylogeny, as well as with the serotype distribution of the genomes.

The REBASE (36) (Gold Standard Database) consisted of 3,211 genes, which were clustered using UCLUST (37), and the curated data set of 2,171 genes was used for restriction modification (RM) system analysis of the genomes. The prevalence pattern of RM systems showed a correlation to the phylogenetic clades and serogroup distribution in the ST95 core genome ML phylogeny (Fig. 5). RM systems of particular interest that were observed included M.Eco9001I, S.Eco9281I, and S.Eco9001I.

The genomes belonging to the exclusively *pks*-negative clade harbored M.Eco9001I (except 95N045, which carried the truncated gene). They also carried either one of S.Eco9281I or S.Eco9001I and Eco9001IP when separately analyzed, as the gene encoding the main restriction enzyme subunit was not included in the REBASE (36) Gold Standard Database. O2:H4 and O25:H4, which also comprised *pks*-negatives, did not carry the above-mentioned genes, and O1:H7 *pks*-positives and three O1:H7 *pks*-negatives (95N039, 95N001, and 95N036) that clustered differently from the main O1:H7 *pks-*negative clade, were also observed not to carry these genes (Fig. 5).

RM system patterns of 530 *pks*-positives and 3,583 *pks*-negatives were also analyzed to decipher their prevalence in these genomes and the ones which showed specific prevalence patterns, such as the type I RM systems Eco9001I/9281I and Eco.CFTI and the type III RM system Eco.CFTII described in Table 3. The modification and recognition genes of these RM systems were part of REBASE (36), while their cognate restriction subunit gene sequences (Eco9001IP/9281IP, Eco.CFTIP, and Eco.CFTIIP) were analyzed separately. While analyzing the sequence types and serotypes of the genomes carrying these RM systems, it was observed that the genomes with the complete Eco.CFTI system belonged, interestingly, to the ST73 complex, but showed no serogroup specificity.

**TABLE 3:** Comparison of prevalence of selected RM systems among *pks-*positive and *pks-*negative genomes

RM system	Genes	Prevalence [no. (%)] among *pks* positives (*n* = 530)	Prevalence [no. (%)] among *pks* negatives (*n* = 3,583)
Eco900I/928I (TYPE-I-RM)	*Eco9001P/9281IP*	124 (23.4)	484 (13.5)
	*M.Eco9001I/9281I*	124 (23.4)	252 (7.03)
	*S.Eco9001I/9281I*	43 (8.11)	43 (1.2)
Eco.CFTI (TYPE-I RM)	*Eco.CFTIP*	257 (48.5)	761 (21.23)
	*M.EcoCFTI*	257 (48.5)	761 (21.23)
	*S.EcoCFTI*	156 (29.43)	7 (0.19)
Eco.CFTII (TYPE-III RM)	*Eco.CFTIIP*	159 (30)	4 (0.11)
	*M.EcoCFTII*	157 (29.62)	0 (0)

### *pks* island phylogeny.

**FIG 6:**
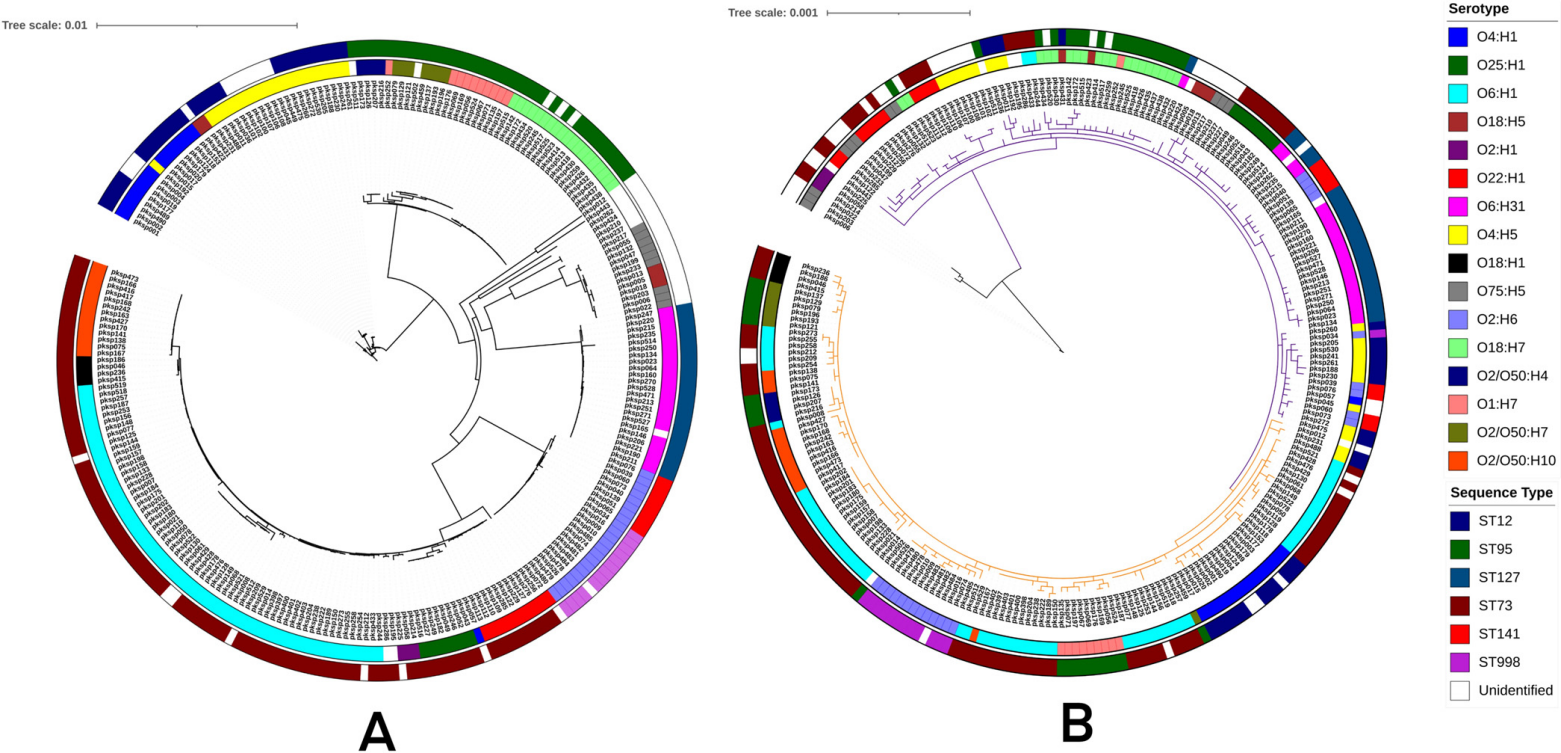
(A) Core genome phylogeny of 247 *pks-*positive genomes constructed using IQTree and visualized using iTOL. (B) Phylogeny of *pks* island sequences from 247 genomes constructed using IQ-TREE and visualized by iTOL. Clade colors depict the three BAPS clusters, i.e., cluster 1 (black), cluster 2 (purple), and cluster 3 (orange). In both panels A and B, the inner ring denotes serotypes and outer ring denotes sequence types of the genomes from which the island was derived; legends for the same have been provided in the figure.

The core genome (number of core genes = 2,579) phylogeny and the *pks* island sequence phylogeny of 247 genomes which contained the *pks* island in a single contig were compared to study the effect of the pattern of evolution of the island sequences with respect to the core genome and the subtype (sequence type and serotype). The core genome phylogeny of the 247 genomes constructed using IQ-TREE (30) showed a clading pattern reflective of the sequence type and serotype, with few exceptions (Fig. 6A). This core genome phylogeny (Fig. 6A) was compared with that of the phylogeny of *pks* island sequences derived from the 247 genomes (Fig. 6B) using Dendroscope (38) as shown in Fig. S3 in the supplemental material. Hierarchical BAPS clustering (31) of the island alignment provided 3 clusters at the first level, which are depicted using different clade colors (black, purple, and orange) in the phylogenetic tree (Fig. 6B). The clading pattern was in agreement with the obtained BAPS clusters. Cluster 1 (black clade) consisted of only 6 genomes, which formed a distinct clade compared to cluster 2 and cluster 3, which comprised the rest of the genomes analyzed. The islands did not show any clustering pattern reflective of the sequence type of their respective genomes in contrast to the core genome phylogeny, except for the *pks* island of genomes from ST998, which were found to cluster together. This lack of concordance with the core genome clustering pattern could indicate that HGT could possibly be the mode of transfer of the island. Islands from ST12, ST73, and ST95 genomes were found to display an intermixed pattern, indicating the possibility of HGT of the island across sequence types (Fig. 6B). The bootstrap support values of the major clades of the core genome phylogeny of 247 *pks-*positive genomes (Fig. 6A) ranged from 98.6% to 100%, and that of the *pks* island phylogeny (Fig. 6B) ranged from 84.6% to 100%.

10.1128/mBio.03634-20.3 FIG S3 Comparison of the core genome phylogeny of 247 *pks*-positive genomes (first panel) along with their *pks* island sequence phylogeny (second panel) using the “connect taxa” functionality of Dendroscope. Download FIG S3, TIF file, 1.2 MB. Copyright © 2021 Suresh et al. 2021 Suresh et al. https://creativecommons.org/licenses/by/4.0/ This content is distributed under the terms of the Creative Commons Attribution 4.0 International license.

## DISCUSSION

The colibactin-producing *pks* island found in certain members of Enterobacteriaceae is emerging as an important virulence marker in the progression of CRC, meningitis, and septicemia (9). Several studies have described the role of colibactin in CRC (19, 39–41), including the synergy between host cells and microbiota in CRC progression (41), making the genotoxin an important virulence factor that requires urgent attention owing to its clinical implications. The *pks* island shows wide distribution among neonatal E. coli K1 isolates and was observed to have a major role in the fully virulent phenotype of the bacteria in a neonatal systemic infection model (12). In the present study, high-throughput phylogenomic comparison of *pks* island-harboring E. coli genomes from the in-house culture collection and publicly available ones from the NCBI were used to draw insights into the island’s acquisition and evolution. The in-house genome collection (*n* = 23) was a part of a previous study from our group, where the isolates linked to a clinical setting from Pune, India, were subjected to epidemiological investigation and characterization of virulence and resistance attributes (26). Whole-genome-based virulome and resistome analysis revealed that the in-house *pks-*positive genomes possessed a high number of genes contributing to virulence (Fig. 1) (see Table S3 in the supplemental material). Genes conferring antimicrobial resistance prevalent in the *pks*-positive genomes mostly consisted of efflux pumps, and only a few specific antibiotic resistance determinants were observed (Fig. 2) (see Table S4 in the supplemental material). These findings were in line with the phenotypic observation of reduced antibiotic resistance and increased functional virulence characteristics displayed by the *pks-*positive isolates in our previous study (26) compared to the frequently observed multidrug-resistant *pks-*negative ExPEC clones obtained from Indian population (42–46). Our previous genomic studies on the *pks-*negative ExPEC collection displayed a higher prevalence of specific antibiotic resistance genes and a relatively lower prevalence of virulence genes (45–47) compared to those in our current analysis of the *pks-*positive genomes. Notably, all of the *pks*-positive genomes harbored the *bacA* gene, which is involved in resistance against the antibiotic, bacitracin (48), and the gene(s) *pmrE*, *pmrC*, and *pmrF*, involved in the binding of polymyxin (49). The large virulence gene repertoire in *pks-*positive isolates is consistent with the previous report based on PCR-based observations on bacteremia isolates (50), implying its clinical significance. Adhesins and type VI secretion systems showed abundance, and there was an increased prevalence of genes belonging to different siderophore production systems (Fig. 1), in concordance with the phenotypic observations of siderophore production assay from our earlier study (26) and other reports, which indicate potential associations between the *pks* island and iron acquisition systems (51). A previous study reported that the *pks* island encoded peptidase ClbP is involved in the genotoxin activation as well as renders antimicrobial activity either through microcins (Mcc) biosynthesis or secretion independently, or in cooperation with glucosyltrasferase, thus reflecting the crucial co-selection of these islands in the evolution of pathogenic phylogroup B2 (16). In a recent study, microcin, salmochelin, and colibactin have also been indicated as a triad that could potentially provide a selective advantage for bacterial colonization in the rectal reservoir with minimal genetic cost (52). The abundance of the siderophore systems like yersiniabactin, enterobactin, salmochelin, and *chuASTUVWXY* genes, along with the *pks* island, could potentially play a role in the successful colonization and persistence of these isolates. Virulence factor profiling also showed an increased prevalence of the hemolysin system (*hly*) in *pks-*positive isolates (Fig. 1), the association of which was indicated previously as a risk factor for colorectal cancer (53).

The study was further expanded to screen 4,090 genomes of E. coli obtained from NCBI, out of which the *pks* island was detected in 507 genomes, in addition to the 23 in-house genomes. The 530 *pks*-positive genomes were further subjected to various *in silico* typing methods to identify distribution patterns among various E. coli subtypes (Table 1). All of the ST73 E. coli isolates were observed to harbor the *pks* island, in contrast to the most prevalent and highly successful ExPEC pandemic clone ST131 data set, which was notably completely *pks*-negative. It is also interesting to note that the prominent STs with *pks* island-positive genomes, i.e., ST73 and ST95, have been previously reported to show low antibiotic resistance (2, 54, 55), which, along with our previous study (26) and Comprehensive Antibiotic Resistance Database (CARD)-based genome analysis in the current study could indicate the association of the *pks* island with isolates having a reduced antimicrobial resistance profile. Phylogrouping showed a strong association of the *pks* island with the B2 phylogroup, in accordance with previous reports (15, 23, 50, 56, 57), as well as with our previous study (26).

ST95 is a successful ExPEC clonal complex that displays functional virulence properties of host adhesion, invasion, biofilm, and serum resistance (58), and it has clinical implications in urinary tract infection and newborn meningitis, while also being a predominant avian and companion animal pathogen (2). The ST95 data set was used as a model for studying the *pks* island, as it was the only sequence type which carried a comparable number of *pks-*positive and *pks*-negative genomes. A total of five clades were obtained (Fig. 3), which were comparable to a previous study about the analysis of STc95 genomes that identified 5 subgroups within the STc95 complex (59). Clades I, II, III, IV, and V of the core genome phylogeny in our study (Fig. 3) showed correspondence to subgroups C, E, B, D, and A, respectively, based on the similar serotype and *fimH* type (59) (clade III additionally carried O2:H5, O1:H7, O1:H1, and O2:H7 genomes in small numbers). The prevalence pattern of the *pks* island sequence was in line with the previously observed prevalence of the *clbB* gene in the same study (59). Differential clading patterns observed in ST95 core genome phylogeny with separate positive, negative, and mixed clusters indicate the potential role of the core genome in HGT and integration of the island (Fig. 3). Core intergenic region-based phylogeny showed a clading pattern more reflective of *pks* island carriage (Fig. 4). A previous study demonstrated that the patterns of polymorphism of the intergenic region *o454-nlpD* displayed concordance with the phylogenetic background, as well as with some important virulence-associated genes in E. coli (60). Core intergenic region substitutions were previously described to show association with the acquisition of an accessory genome in ST131 E. coli (61), and the analysis of ST95 genomes with respect to the *pks* island exhibited a similar pattern. Although most of the clustering patterns of the ST95 core genome phylogeny reflected the serotypes, O1:H7 showed a peculiar distribution into different clades containing *pks-*positive and *pks*-negative genomes, and they also had a distinct *fimH* type (Fig. 4). Scoary (33) was used for pangenome-wide analysis of accessory genes, where the positive and mixed clusters were used as a combined data set (clades I, II, III, and V) to compare with genomes belonging to a completely *pks-*negative clade (clade IV) and the genes that showed differential enrichment among the groups listed in Table 2. It was interesting to note that the genes *idnODRKT* belonging to the subsidiary system for l-idonic acid catabolism, which may provide a metabolic advantage for colonization (62), were present in all genomes belonging to the *pks-*positive and mixed cluster, while being completely absent in the members of the exclusively *pks*-negative clade (clade IV). The type 1 restriction enzyme R protein *hsdR* and the DNA methylase *hsdM* were observed to be present only among the genomes belonging to the exclusively *pks*-negative clade (Table 2).

As RM systems are shown to be involved in the regulation of HGT and recombination (63), their prevalence was studied among *pks-*positive and *pks*-negative data sets as a preliminary analysis to determine their putative role in transfer or incompatibility of the acquisition of the *pks* island. A previous study has indicated the potential role of restriction modification systems in the acquisition of resistance plasmids in ST95 O1:H7 isolates (64). Since the *pks* island showed clade-specific distribution patterns within the ST95 core genome phylogenetic tree, the tree topology was compared with its RM system prevalence data as a model to study the RM system diversity and finer distribution pattern (Fig. 5). The analysis is limited to the RM systems in the curated Gold Standard Database of REBASE and their selected cognate restriction enzyme subunit counterparts of the systems. When overlaid with the core genome phylogeny, the topology of RM prevalence pattern showed relation to the subclades reflective of their serotypes (Fig. 5). This observation is similar to the results from a previous study describing the methyl transferase diversity among ST131 E. coli isolates in which the RM system profiles were observed to show relation to their phylogenetic clusters (65). Another study in Burkholderia pseudomallei showed the clade-specific complement of the RM system, which potentially led to the clade-specific patterns in the DNA methylome (66). The population structure of Neisseria meningitidis was also observed to coincide with its RM system distribution, suggesting a role of RM systems as a barrier in DNA exchange, driving the formation of distinct phylogenetic lineages (67). Similar sublineage correlations based on serovars and phylogenetic clading of genomes with identical RM profiles were observed in a previous study involving Salmonella enterica (68). Based on this evidence and our observations, we hypothesize that the RM system profiles of the isolates might have a potential role in shaping the phylogenetic lineages and guiding the DNA exchange, thus playing a role in the horizontal acquisition of the genomic island. Notably, in the analysis of the RM system profile in the entire *pks-*positive and *pks-*negative data sets, the type III RM system Eco.CFTII showed a higher prevalence in *pks-*positive genomes than in the *pks*-negative genomes (Table 3). However, the limitation of a small number of curated candidates available for RM system analysis is to be noted, and careful interpretation is mandated. Based on these preliminary observations from prevalence analysis of RM systems among *pks*-positive and *pks-*negative genomes, further studies on their probable role in the acquisition and maintenance of the mobile genetic elements will be required.

The phylogeny of the *pks* island, in contrast to core genome phylogeny, revealed its mixed distribution among various sequence types and serotypes (except in certain groups) indicative of a probable frequent HGT across the sequence types (Fig. 6; see also Fig. S3 in the supplemental material). This observation is in line with the evidence from a previous study in which the comparison between phylogenetic trees of the core genome and the *pks* island sequences within the ECOR collection displayed different clustering patterns indicative of the transmission of the island to be horizontal and not vertical (25). This, along with other observations of prevalence patterns, demonstrated that certain sequence types of E. coli, such as ST73, ST95, and ST12, show increased capability to acquire the island, and frequent horizontal exchanges of the island could occur across these subtypes.

In conclusion, our study is perhaps the first one to perform large-scale, whole-genome-based investigations with respect to the distribution of the *pks* island(s) among different E. coli populations and the consequent evolutionary relationships. The preferential distribution pattern of the *pks* (encoded genotoxin)-harboring E. coli was studied using different computational methods of subtyping. These observations may be able to provide support to the diagnostic systems or health care modalities aimed at understanding the clinical implications of the potential genotoxic nature of *pks*-positive isolates. The *pks* island phylogeny indicated horizontal acquisition/transmission and the possibility of exchange between compatible E. coli subtypes. Investigation of the ST95 model data set revealed a higher prevalence of the *pks* island within specific serotypes and CH types, pointing at the role of HGT and finer evolution within a particular ST. The core genome and core intergenic region phylogeny were used to gain a comprehensive understanding of the clade-specific pattern of distribution of the island, which is otherwise a part of the accessory genome. Further studies on the potential role of RM systems in shaping the lineages and driving the acquisition of the island among compatible isolates needs to be performed at a higher resolution in order to gain interesting insights into the HGT and evolution of virulence in pathogenic E. coli.

## MATERIALS AND METHODS

### Ethics statement.

All of the E. coli isolates that are newly unraveled here were originally isolated as part of our previous studies, as mentioned. Cultures and DNA preparations were handled as per standard biosafety guidelines for E. coli and within the ambit of available permissions.

### Whole-genome sequencing, assembly, and annotation.

Genomic DNA of 25 *pks-*positive (in-house) E. coli isolates (originally cultured and maintained by S.J. and her colleagues from Dr, D. Y. Patil University Hospital, Pune, India), which were characterized in our previous study (26), were isolated and purified of any RNA contamination using a Qiagen DNeasy blood and tissue kit (Qiagen, Germany) and sequenced using the Illumina MiSeq platform (69, 70). The paired-end reads were subjected to quality control using NGS QC Toolkit (71), trimmed using FastX-Trimmer (http://hannonlab.cshl.edu/fastx_toolkit/), and further assembled *de novo* using SPAdes Genome Assembler (v3.6.1) (72). Assembly statistics were obtained using QUAST (73). Two out of the 25 isolates were discarded from further analysis due to poor quality. The contigs were further ordered and scaffolded using C-L-Authenticator (74), using the E. coli ATCC 25922 complete genome as a reference, and the scaffolds were annotated using Prokka (75). Genome statistics were gleaned using Artemis (76), and sequence types of the isolates were identified using an *in silico* MLST pipeline using in-house scripts (47, 77) and the publicly available MLST pipeline (https://github.com/tseemann/mlst), which uses the PubMLST database (https://pubmlst.org/) (78). ECTyper (https://github.com/phac-nml/ecoli_serotyping) was used to perform *in silico* serotyping of the genomes.

### Analysis of the genomes for the resistance and virulence determinants.

Amino acid sequence files from annotated genomes were used to determine the resistance and virulence genes by performing BLASTp (79) against the Comprehensive Antibiotic Resistance Database (80) and Virulence Factor Database (28), respectively. A percentage identity of 70% and a query coverage of 75% were used as thresholds while analyzing the genomes for the presence of the respective genes. The heat plots depicting the presence-absence status of the genes were generated using the *gplots* (https://github.com/talgalili/gplots) package of R. The virulence and resistance profiles of 23 in-house *pks*-positive genomes were also compared with those of 23 in-house *pks-*negative genomes using the methodology mentioned above (accession IDs are listed in Table S6 in the supplemental material).

### Whole-genome comparative analysis and visualization.

The complete genome of E. coli IHE3034 (GenBank accession number CP001969.1) was used as the reference genome, and the assembled genomes of in-house isolates harboring the *pks* islands were compared to the reference *pks*-positive genomes using BLAST Ring Image Generator (BRIG) (27) to determine their genetic relatedness, with upper and lower identity thresholds of 70% and 50%, respectively. The annotation of phages in the reference genome was performed using the PHAST server (81), and the coordinates of the loci of the detected phages were plotted on the image. The coordinates of the *pks* island were also annotated in the BRIG image.

The trimmed, filtered reads of the genomes were mapped and aligned to the reference sequence of the *pks* island along with flanking regions obtained from NCBI (GenBank accession number AM229678.1) using SAMtools (82) and Bowtie 2 (83). The mapped reads were assembled *de novo* using SPAdes (72), and the reconstructed island sequences were obtained and then further subjected to BRIG (27) using the complete *pks* island sequence as the reference to visualize the integrity of the island. The upper and lower identity thresholds used in the analysis were 70% and 50%, respectively.

### Identification of *pks* island-containing genomes in the public domain.

E. coli genomes were downloaded from the public database using in-house scripts, and the genomes with size greater than 4.8 Mb and with fewer than 200 contigs each, were used for the study. A total of 3,784 draft and 306 complete genomes were selected after curation as the final database for further downstream analysis. The complete *pks* island along with flanking regions of E. coli IHE3034 and its coding sequences were obtained from NCBI as the reference sequence of the genomic island (GenBank accession number AM229678.1). The genomes were screened for the presence of *pks* island genes obtained from the above references using BLASTn (79) with identity and query coverage thresholds of 85%.

### Genome annotation, *in silico* MLST and phylogrouping.

The genomes of both NCBI and in-house isolates were subjected to annotation using Prokka software (75). All the genomes were subjected to *in silico* phylogrouping and multilocus sequence typing (MLST) to determine the sequence types using an *in silico* MLST pipeline that harnessed in-house scripts (47, 77) and the MLST pipeline (https://github.com/tseemann/mlst), which uses the PubMLST database (https://pubmlst.org/) (78). ECTyper (https://github.com/phac-nml/ecoli_serotyping) was used to perform the *in silico* serotyping of the genomes.

### ST95 pangenome analysis.

ST95 was used as a model data set to study the distribution and evolutionary pattern of the *pks* island due to the availability of both *pks-*positive (*n* = 110) and *pks*-negative genomes (*n* = 49). The pangenome analysis of 159 genomes from ST95 after annotation using Prokka (75) was performed using Roary (84) with identity and *E* value cutoffs of 85% and 0.00001, respectively, for the determination of orthologous gene clusters. Genes which were shared by all the 159 isolates, which constitute the core genome, and the core genes were subjected to COG classification using eggNOG (29), and the COG groups were tabulated. The genomes were also analyzed using CHTyper (85) for the *in silico* determination of CH types based on *fumC* and *fimH* alleles.

### ST95 core genome phylogeny.

The core genes determined using Roary were subjected to nucleotide alignment using PRANK (86), and the resultant core genome alignment was further subjected to trimAl (87) (using -strict flag) for the trimming and refinement of the alignment. The alignment was also used as an input for hierBAPS (31) to perform hierarchical clustering based on sequence variations using Bayesian methods. The refined alignment was then subjected to IQ-TREE (30) with ModelFinder (88) to optimize the best nucleotide substitution model to construct a maximum-likelihood phylogenetic tree with 500 bootstrap replicates. The resultant core genome based maximum-likelihood phylogenetic tree was subjected to ClonalFrameML (89) to remove recombination regions and was visualized using interactive Tree Of Life (iTOL) (90). The branches were color coded according to the presence/absence of the *pks* island, and BAPS cluster, serogroup, and CH type information were also annotated in the tree using data strips. In addition, a core genome phylogenetic tree including an outgroup, ED1a (NCBI assembly number GCA_000026305.1), was also constructed using the methodology mentioned above. A pangenome-wide association study comparing the genomes belonging to *pks*-positive and mixed clades with genomes belonging to the exclusively *pks-*negative clade (as identified in core genome-based phylogeny) was performed using Scoary (33) with the help of the gene_presence_absence.csv output file of Roary (84). The *pks-*positive genomes (*n* = 110) and *pks-*negative genomes belonging to the mixed clade (*n* = 20) were grouped together and designated with trait value “1” and the *pks*-negative genomes forming the exclusively *pks-*negative clade (*n* = 29) were designated with trait value “0” in the Scoary (33) input. The prevalence of the differentially enriched genes between the two groups was determined across the *pks-*positive (*n* = 530) and *pks*-negative (*n* = 3583) genomes using BLASTn (79) with identity and query coverage thresholds of 85%.

### ST95 IGR phylogeny.

The GFF files that were derived from the annotation of 159 ST95 genomes using Prokka (75) and the gene presence-absence file obtained from pangenome analysis by Roary (84) were used to perform the intergenic region analysis using Piggy (35). The intergenic regions (IGRs) that were shared by all the genomes (core IGRs) were extracted and aligned using Prank (86), followed by trimming using trimAl (87) to refine the alignment by removing spurious and poorly aligned regions. IQ-TREE (30) was employed along with ModelFinder (88) (-MFP flag) for construction of IGR phylogeny with 500 bootstrap replicates, followed by ClonalFrameML (89) to produce a maximum-likelihood phylogeny of the core intergenic regions of ST95 genomes. The resultant phylogenetic tree was visualized using iTOL (90), with serogroup and CH type information of the isolates, which was previously obtained, labeled as data strips.

### RM system analysis.

The restriction modification (RM) gene profiling of the E. coli genomes was performed using the REBASE Gold Standard Database (36). The REBASE database was clustered using UCLUST (37) with an identity threshold of 90%. This curated database was used for the detection of RM systems in E. coli genomes. A BLASTn search was performed against genomes with identity and query coverage thresholds of 85%. In order to determine the pattern of distribution of RM systems among *pks-*positive and *pks*-negative genomes, BLAST analysis of curated RM systems database was performed against the data sets, namely, ST95 genomes (*n* = 159), *pks-*positive genomes (*n* = 530), and *pks-*negative genomes (*n* = 3,583). In cases where the genomes were observed to carry the modification and recognition subunits, the sequences of their cognate restriction enzymes obtained from REBASE were also separately analyzed, if they were not already included in the gold-standard database.

### *pks* island phylogeny.

The *pks-*positive genomes were subjected to standalone BLAST analysis against *pks* island sequence, and the genomes with identity and query coverage of greater than 95% and 85%, respectively, for the *pks* island were used to obtain genome sequences harboring the *pks* island within a single contig. The core genome phylogeny of these selected genomes (*n* = 247) was constructed per the methodology mentioned in the previous sections. The *pks* island sequences from these genomes were extracted from the locus information of BLAST outputs using the extract-align program from EMBOSS (http://emboss.sourceforge.net/apps/cvs/emboss/apps/extractalign.html) and in-house scripts to handle reverse complements. The island sequences were aligned using PRANK (86), and trimAl (used with -strict flag) (87) was used to refine the alignment. Bayesian analysis of population structure (BAPS) (31) clustering of the alignment was performed for the sequences, and IQ-TREE (30) with 1,000 bootstrap replicates was used for the construction of a maximum-likelihood phylogeny with ModelFinder enabled (88) and visualized using iTOL (90), along with annotations for sequence type and serotype information. The two phylogenetic trees were also compared using the “connect taxa” functionality of Dendroscope (v3.7.3) (38).

### Data availability.

The genome sequence data generated as a part of this study are deposited in NCBI under the BioProject identifier PRJNA667681. Accession numbers of the individual genomes described here are as follows: JADBJB000000000, JADBJA000000000, JADNRJ000000000, JADBIZ000000000, JADBIY000000000, JADBIX000000000, JADBIW000000000, JADBIV000000000, JADBIU000000000, JADBIT000000000, JADBIS000000000, JADBIR000000000, JADBIQ000000000, JADBIP000000000, JADBIO000000000, JADBIN000000000, JADBIM000000000, JADBIL000000000, JADBIK000000000, JADBIJ000000000, JADBII000000000, JADBIH000000000, and JADBIG00000000 (see Table S2 in the supplemental material for further details).
