# Supplementary figures and images for: Evolutionary Dynamics Based on Comparative Genomics of Pathogenic Escherichia coli Lineages Harboring Polyketide Synthase (pks) Island
Source: mBio. 2021 Mar 2;12(1):e03634-20. doi: 10.1128/mBio.03634-20 (PMC8545132; doi:10.1128/mBio.03634-20)

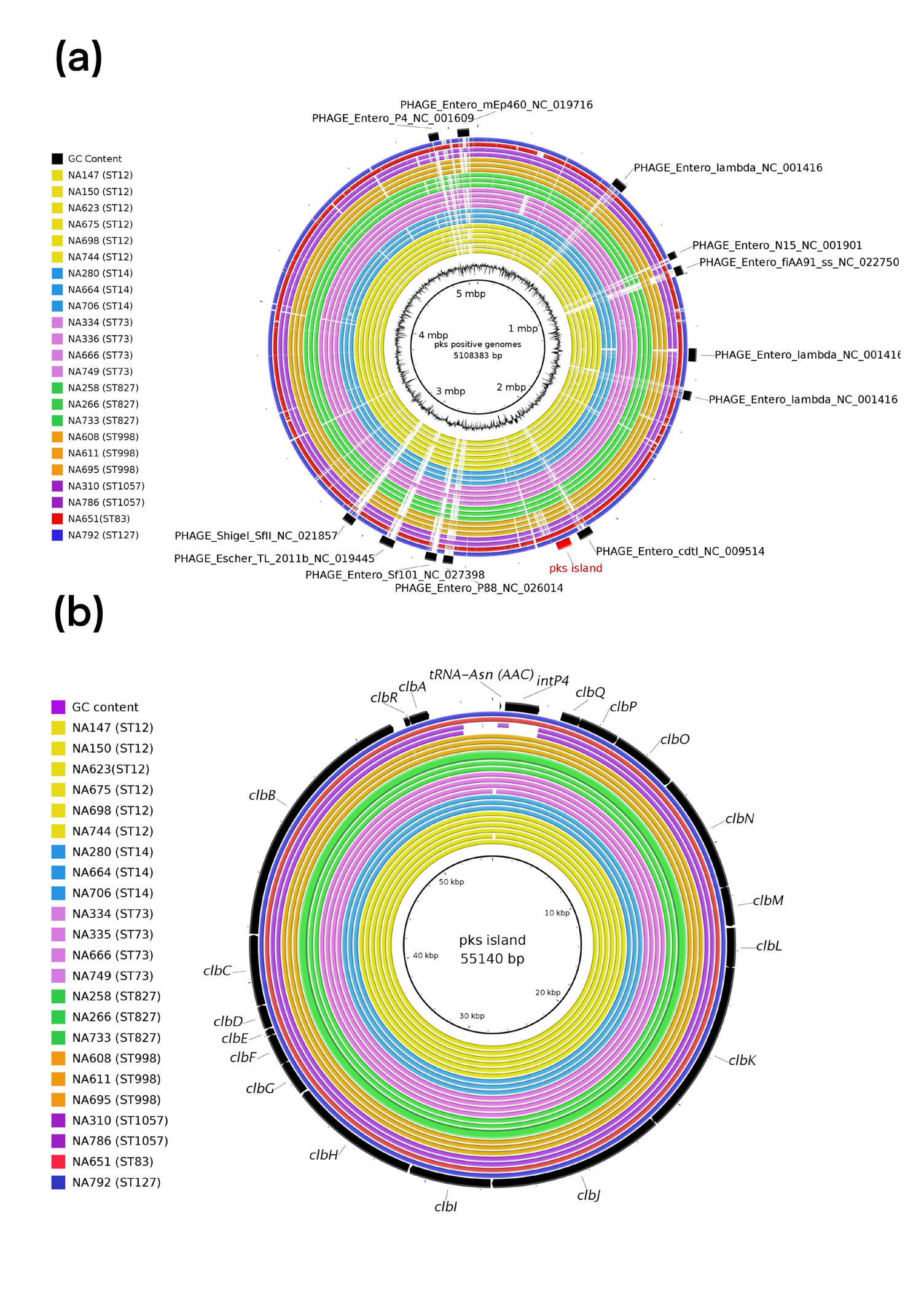

Supplement: FIG S1 [file mbio.03634-20-sf001.tif]

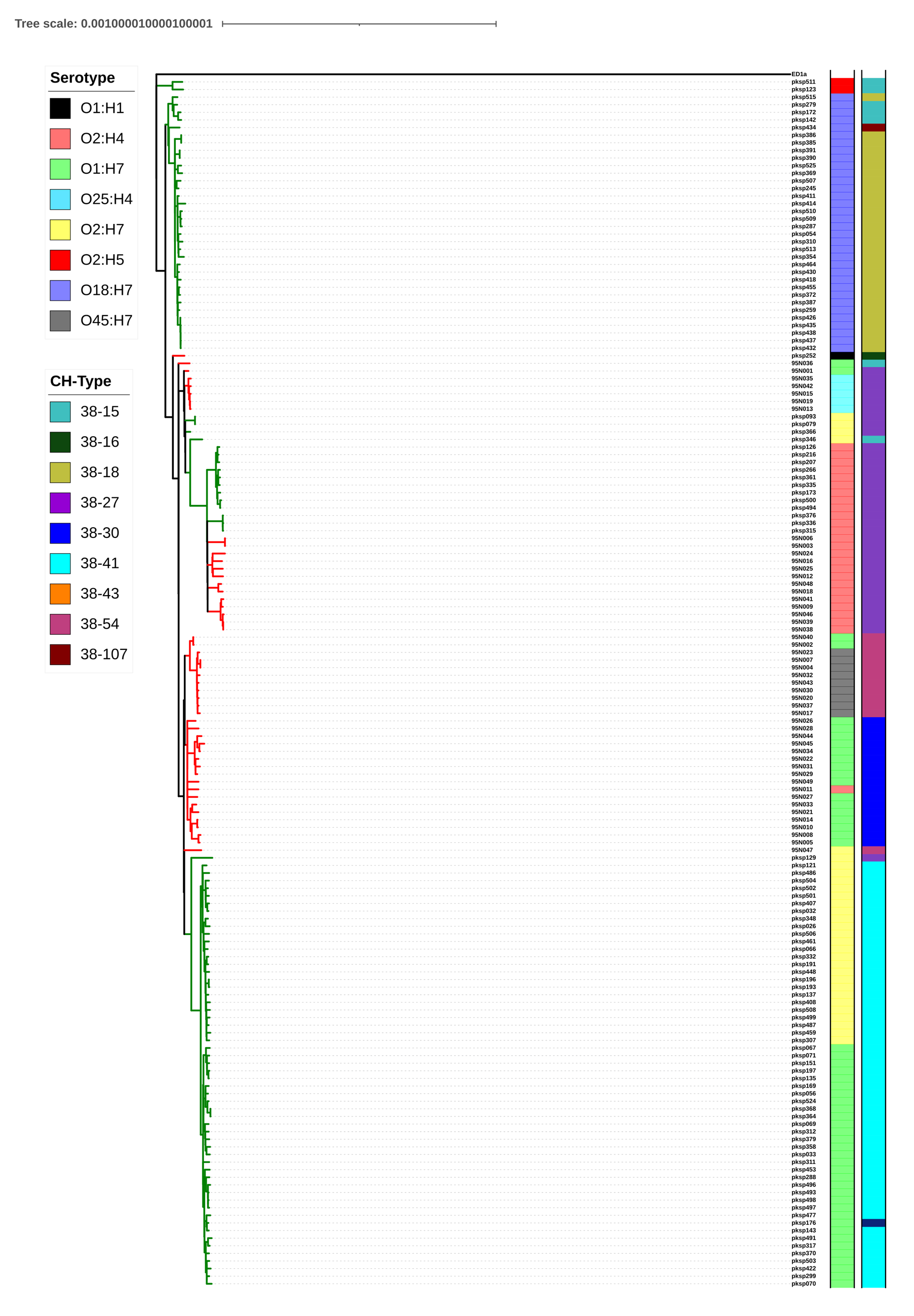

Supplement: FIG S2 [file mbio.03634-20-sf002.tif]

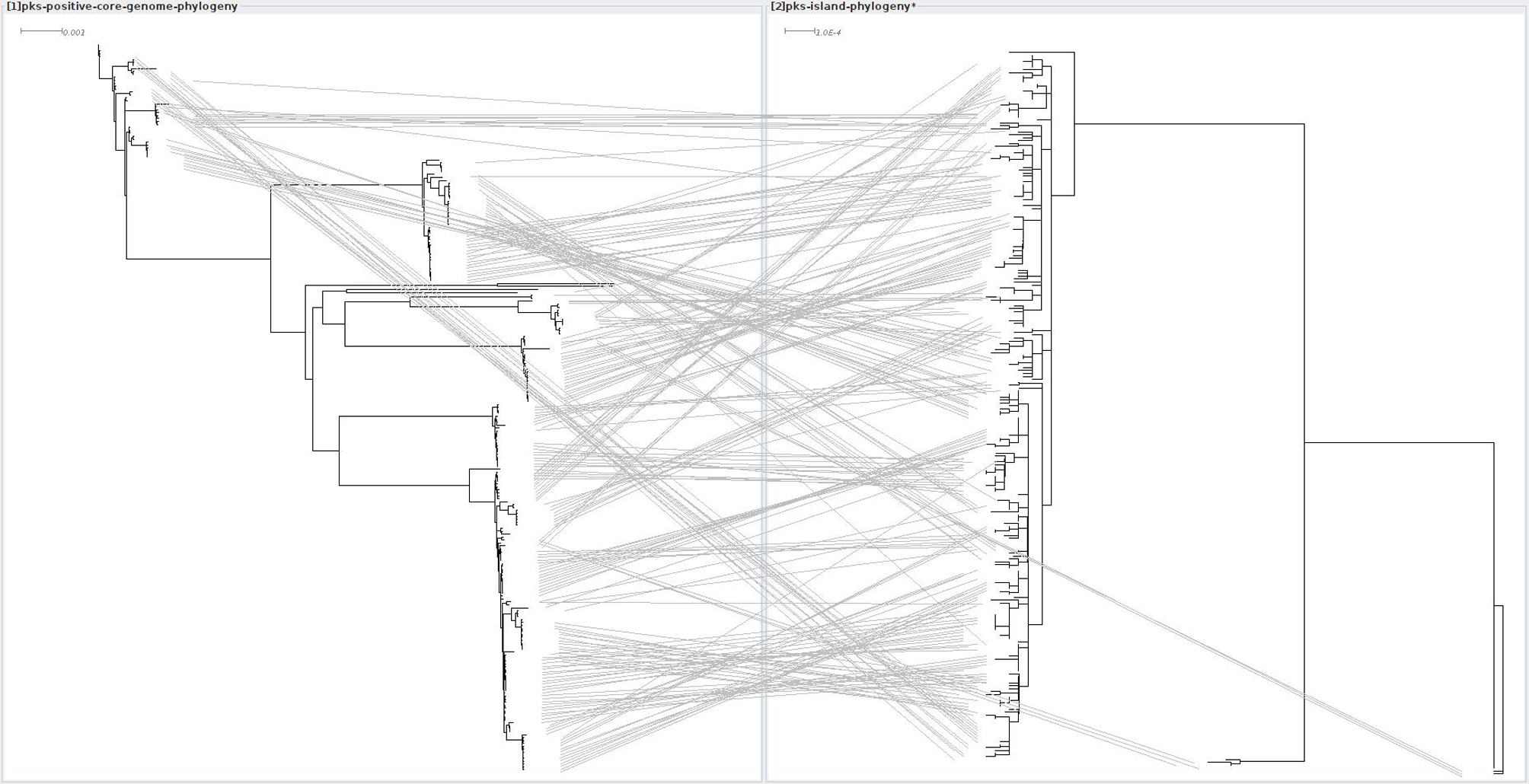

Supplement: FIG S3 [file mbio.03634-20-sf003.tif]
