# Supplementary material for: Evolutionary Dynamics Based on Comparative Genomics of Pathogenic Escherichia coli Lineages Harboring Polyketide Synthase (pks) Island
Source: mBio. 2021 Mar 2;12(1):e03634-20. doi: 10.1128/mBio.03634-20 (PMC8545132; doi:10.1128/mBio.03634-20)
Supplement: TABLE S1 [file mbio.03634-20-st001.pdf]

**Table S1**: Assembly and scaffolding statistics of the *pks* positive genomes

| Isolate | Avg. Genome coverage | No. of filtered contigs | Number of scaffolds | Total bp |
|---|---|---|---|---|
| **NA147** | 89.077 | 104 | 93 | 5001103 |
| **NA150** | 59.886 | 100 | 97 | 5007721 |
| **NA258** | 44.567 | 179 | 174 | 5163691 |
| **NA266** | 76.36 | 131 | 108 | 5212596 |
| **NA280** | 63.49 | 150 | 137 | 5274649 |
| **NA310** | 58.63 | 171 | 161 | 5269727 |
| **NA334** | 51.42 | 162 | 150 | 5159446 |
| **NA336** | 61.98 | 159 | 137 | 5259602 |
| **NA608** | 183.89 | 78 | 51 | 5178873 |
| **NA611** | 47.284 | 82 | 71 | 5107459 |
| **NA623** | 71.59 | 178 | 168 | 5200798 |
| **NA651** | 76.34 | 63 | 53 | 5177187 |
| **NA664** | 63.17 | 183 | 169 | 5306725 |
| **NA666** | 65.95 | 132 | 118 | 5105662 |
| **NA675** | 49.8 | 98 | 88 | 5159592 |
| **NA695** | 69.16 | 86 | 74 | 5135536 |
| **NA698** | 62.03 | 176 | 162 | 5266727 |
| **NA706** | 62.46 | 149 | 136 | 5315252 |
| **NA733** | 70.07 | 117 | 106 | 5226616 |
| **NA744** | 79.51 | 112 | 95 | 5195215 |
| **NA749** | 80.54 | 144 | 128 | 5221527 |
| **NA786** | 78.78 | 157 | 140 | 5395297 |
| **NA792** | 82.78 | 78 | 61 | 4999821 |
