# Supplementary material for: Evolutionary Dynamics Based on Comparative Genomics of Pathogenic Escherichia coli Lineages Harboring Polyketide Synthase (pks) Island
Source: mBio. 2021 Mar 2;12(1):e03634-20. doi: 10.1128/mBio.03634-20 (PMC8545132; doi:10.1128/mBio.03634-20)
Supplement: TABLE S2 [file mbio.03634-20-st002.pdf]

Table S2: Genome characteristics of *pks* positive isolates

Isolate	No. of CDS	Avg. CDS length	Coding %	GC%	No. of rRNAs	No. of tRNAs	ST	Serotype	Accession Number
**NA147**	4825	908	87.6	50.49	9	73	ST12	O4:H1	JADBJB000000000
**NA150**	4810	911	87.5	50.5	9	73	ST12	O4:H1	JADBJA000000000
**NA258**	5042	891	87	50.59	8	69	ST827	O4:H1	JADNRJ000000000
**NA266**	5098	892	87.2	50.46	9	85	ST827	O4:H1	JADBIZ000000000
**NA280**	5164	886	86.8	50.49	14	89	ST14	O18:H5	JADBIY000000000
**NA310**	5194	883	87.1	50.53	9	78	ST1057	O75:H5	JADBIX000000000
**NA334**	4995	893	86.5	50.31	11	68	ST73	O6:H1	JADBIW000000000
**NA336**	5173	882	86.7	50.39	10	69	ST73	O6:H1	JADBIV000000000
**NA608**	5013	905	87.6	50.52	10	74	ST998	O2:H6	JADBIU000000000
**NA611**	4917	911	87.7	50.54	10	68	ST998	O2:H6	JADBIT000000000
**NA623**	5081	889	86.9	50.45	9	78	ST12	O4:H5	JADBIS000000000
**NA651**	5038	900	87.6	50.41	10	73	ST83	O6:H5	JADBIR000000000
**NA664**	5219	883	86.8	50.52	13	77	ST14	O18:H5	JADBIQ000000000
**NA666**	4956	894	86.8	50.44	12	74	ST73	O6:H1	JADBIP000000000
**NA675**	5035	896	87.4	50.52	13	78	ST12	O4:H1	JADBIO000000000
**NA695**	4934	910	87.5	50.52	12	74	ST998	O2:H6	JADBIN000000000
**NA698**	5168	886	87	50.5	9	76	ST12	O4:H1	JADBIM000000000
**NA706**	5223	886	87	50.5	10	86	ST14	O18:H5	JADBIL000000000
**NA733**	5106	893	87.3	50.5	9	75	ST827	O4:H1	JADBIK000000000
**NA744**	5069	893	87.1	50.4	10	73	ST12	O4:H1	JADBIJ000000000
**NA749**	5081	892	86.8	50.34	8	77	ST73	O6:H1	JADBII000000000
**NA786**	5347	881	87.3	50.5	11	81	ST1057	O75:H5	JADBIH000000000
**NA792**	4801	913	87.7	50.47	11	78	ST127	O6:H31	JADBIG00000000
