# Supplementary material for: Evolutionary Dynamics Based on Comparative Genomics of Pathogenic Escherichia coli Lineages Harboring Polyketide Synthase (pks) Island
Source: mBio. 2021 Mar 2;12(1):e03634-20. doi: 10.1128/mBio.03634-20 (PMC8545132; doi:10.1128/mBio.03634-20)
Supplement: TABLE S5 [file mbio.03634-20-st005.pdf]

Table S5: Accession numbers and isolate names of 530 *pks* positive NCBI genomes used in the study

(Genomes denoted in italics (pksp001 to pksp023) represents the newly sequenced genomes for the study. Genomes belonging to ST95 (n=110) are denoted with * in their IDs.)

ID	Isolate	Assembly	ID	Isolate	Assembly
pksp001	*NA147*	JADBJB000000000	pksp024	83972	GCA_000159295.1
pksp002	*NA150*	JADBJA000000000	pksp025	MS 45-1	GCA_000164295.1
pksp003	*NA258*	JADNRJ000000000	pksp026*	MS 110-3	GCA_000164415.1
pksp004	*NA266*	JADBIZ000000000	pksp027	MS 153-1	GCA_000164435.1
pksp005	*NA280*	JADBIY000000000	pksp028	MS 200-1	GCA_000164535.1
pksp006	*NA310*	JADBIX000000000	pksp029	MS 185-1	GCA_000164575.1
pksp007	*NA334*	JADBIW000000000	pksp030	MS 60-1	GCA_000164595.1
pksp008	*NA336*	JADBIV000000000	pksp031	NC101	GCA_000179795.1
pksp009	*NA608*	JADBIU000000000	pksp032*	H263	GCA_000190915.1
pksp010	*NA611*	JADBIT000000000	pksp033*	H397	GCA_000241975.1
pksp011	*NA623*	JADBIS000000000	pksp034	9.1649	GCA_000194475.2
pksp012	*NA651*	JADBIR000000000	pksp035	LCT-EC106	GCA_000259695.1
pksp013	*NA664*	JADBIQ000000000	pksp036	J96	GCA_000295775.2
pksp014	*NA666*	JADBIP000000000	pksp037	LCT-EC52	GCA_000331615.1
pksp015	*NA675*	JADBIO000000000	pksp038	LCT-EC59	GCA_000317395.1
pksp016	*NA695*	JADBIN000000000	pksp039	KTE15	GCA_000350745.1
pksp017	*NA698*	JADBIM000000000	pksp040	KTE16	GCA_000350765.1
pksp018	*NA706*	JADBIL000000000	pksp041	KTE39	GCA_000350865.1
pksp019	*NA733*	JADBIK000000000	pksp042	KTE187	GCA_000350945.1
pksp020	*NA744*	JADBIJ000000000	pksp043	KTE188	GCA_000350965.1
pksp021	*NA749*	JADBII000000000	pksp044	KTE189	GCA_000350985.1
pksp022	*NA786*	JADBIH000000000	pksp045	KTE191	GCA_000351005.1
pksp023	*NA792*	JADBIG00000000	pksp046	KTE201	GCA_000351045.1

ID	Isolate	Assembly
pksp047	KTE205	GCA_000351085.1
pksp048	KTE206	GCA_000351105.1
pksp049	KTE214	GCA_000351205.1
pksp050	KTE220	GCA_000351245.1
pksp051	KTE224	GCA_000351265.1
pksp052	KTE230	GCA_000351305.1
pksp053	KTE53	GCA_000351485.1
pksp054*	KTE55	GCA_000351505.1
pksp055	KTE57	GCA_000351545.1
pksp056*	KTE58	GCA_000351565.1
pksp057	KTE60	GCA_000351585.1
pksp058	KTE67	GCA_000351645.1
pksp059	KTE72	GCA_000351665.1
pksp060	KTE86	GCA_000351805.1
pksp061	KTE87	GCA_000351825.1
pksp062	KTE93	GCA_000351845.1
pksp063	KTE169	GCA_000352025.1
pksp064	KTE8	GCA_000352085.1
pksp065	KTE43	GCA_000352225.1
pksp066*	KTE22	GCA_000352265.1
pksp067*	KTE59	GCA_000352365.1
pksp068	KTE63	GCA_000352385.1
pksp069*	KTE65	GCA_000352405.1
pksp070*	KTE118	GCA_000352545.1
pksp071*	KTE123	GCA_000352565.1
pksp072	KTE141	GCA_000352645.1
pksp073	KTE183	GCA_000352905.1
pksp074	KTE207	GCA_000353005.1

ID	Isolate	Assembly
pksp075	KTE209	GCA_000353025.1
pksp076	KTE215	GCA_000353065.1
pksp077	KTE218	GCA_000353105.1
pksp078	KTE223	GCA_000353125.1
pksp079*	KTE229	GCA_000353165.1
pksp080	KTE104	GCA_000353185.1
pksp081	KTE106	GCA_000326165.1
pksp082	KTE124	GCA_000326225.1
pksp083	KTE129	GCA_000326265.1
pksp084	KTE131	GCA_000326285.1
pksp085	KTE133	GCA_000326305.1
pksp086	KTE137	GCA_000326325.1
pksp087	KTE145	GCA_000326705.1
pksp088	KTE153	GCA_000326385.1
pksp089	KTE160	GCA_000326405.1
pksp090	KTE167	GCA_000326905.1
pksp091	KTE168	GCA_000326445.1
pksp092	KTE174	GCA_000326605.1
pksp093*	KTE179	GCA_000326485.1
pksp094	KTE180	GCA_000326805.1
pksp095	KTE85	GCA_000326885.1
pksp096	KTE88	GCA_000326625.1
pksp097	KTE97	GCA_000326545.1
pksp098	KTE99	GCA_000326665.1
pksp099	TOP379	GCA_000397225.1
pksp100	TOP382-1	GCA_000397245.1
pksp101	TOP382-2	GCA_000397265.1
pksp102	TOP382-3	GCA_000397285.1

ID	Isolate	Assembly
pksp103	TOP291	GCA_000397305.1
pksp104	TOP293-2	GCA_000397345.1
pksp105	TOP498	GCA_000397405.1
pksp106	TOP550-2	GCA_000397445.1
pksp107	TOP550-3	GCA_000397465.1
pksp108	TOP550-4	GCA_000397485.1
pksp109	TOP2652	GCA_000397625.1
pksp110	TOP2662-1	GCA_000397645.1
pksp111	TOP2662-2	GCA_000397665.1
pksp112	TOP2662-3	GCA_000397685.1
pksp113	TOP2662-4	GCA_000397705.1
pksp114	HM27	GCA_000387825.2
pksp115	HM65	GCA_000387785.2
pksp116	ATCC 25922	GCA_000401755.1
pksp117	KTE182	GCA_000408065.1
pksp118	KTE195	GCA_000408125.1
pksp119	KTE226	GCA_000408285.1
pksp120	KTE89	GCA_000408505.1
pksp121*	HVH 1 (4-6876161)	GCA_000456005.1
pksp122	HVH 2 (4-6943160)	GCA_000456025.1
pksp123*	HVH 3 (4-7276001)	GCA_000456045.1
pksp124	HVH 4 (4-7276109)	GCA_000456065.1
pksp125	HVH 7 (4-7315031)	GCA_000456125.1
pksp126*	HVH 12 (4-7653042)	GCA_000494955.1

ID	Isolate	Assembly
pksp127	HVH 13 (4-7634056)	GCA_000456185.1
pksp128	HVH 16 (4-7649002)	GCA_000456205.1
pksp129*	HVH 19 (4-7154984)	GCA_000456265.1
pksp130	HVH 20 (4-5865042)	GCA_000456285.1
pksp131	HVH 21 (4-4517873)	GCA_000456305.1
pksp132	HVH 26 (4-5703913)	GCA_000456385.1
pksp133	HVH 27 (4-7449267)	GCA_000456405.1
pksp134	HVH 28 (4-0907367)	GCA_000456425.1
pksp135*	HVH 30 (4-2661829)	GCA_000456465.1
pksp136	HVH 31 (4-2602156)	GCA_000456485.1
pksp137*	HVH 35 (4-2962667)	GCA_000456545.1
pksp138	HVH 37 (4-2773848)	GCA_000456565.1
pksp139	HVH 38 (4-2774682)	GCA_000456585.1
pksp140	HVH 39 (4-2679949)	GCA_000456605.1
pksp141	HVH 40 (4-1219782)	GCA_000456625.1
pksp142*	HVH 42 (4-2100061)	GCA_000456665.1
pksp143*	HVH 48 (4-2658593)	GCA_000456765.1

ID	Isolate	Assembly
pksp144	HVH 51 (4-2172526)	GCA_000456785.1
pksp145	HVH 55 (4-2646161)	GCA_000456825.1
pksp146	HVH 56 (4-2153033)	GCA_000456845.1
pksp147	HVH 58 (4-2839709)	GCA_000456865.1
pksp148	HVH 61 (4-2736020)	GCA_000456905.1
pksp149	HVH 68 (4-0888028)	GCA_000456965.1
pksp150	HVH 74 (4-1034782)	GCA_000457045.1
pksp151[*]	HVH 76 (4-2538717)	GCA_000457065.1
pksp152	HVH 77 (4-2605759)	GCA_000457085.1
pksp153	HVH 78 (4-2735946)	GCA_000457105.1
pksp154	HVH 80 (4-2428830)	GCA_000457145.1
pksp155	HVH 86 (4-7026218)	GCA_000494975.1
pksp156	HVH 89 (4-5885604)	GCA_000457265.1
pksp157	HVH 92 (4-5930790)	GCA_000457325.1
pksp158	HVH 95 (4-6074464)	GCA_000457345.1
pksp159	HVH 96 (4-5934869)	GCA_000457385.1
pksp160	HVH 100 (4-2850729)	GCA_000457405.1

ID	Isolate	Assembly
pksp161	HVH 103 (4-5904188)	GCA_000457435.1
pksp162	HVH 107 (4-5860571)	GCA_000457495.1
pksp163	HVH 109 (4-6977162)	GCA_000457515.1
pksp164	HVH 111 (4-7039018)	GCA_000457555.1
pksp165	HVH 112 (4-5987253)	GCA_000457575.1
pksp166	HVH 114 (4-7037740)	GCA_000457615.1
pksp167	HVH 116 (4-6879942)	GCA_000457675.1
pksp168	HVH 117 (4-6857191)	GCA_000457695.1
pksp169[*]	HVH 118 (4-7345399)	GCA_000457715.1
pksp170	HVH 120 (4-6978681)	GCA_000457755.1
pksp171	HVH 125 (4-2634716)	GCA_000457815.1
pksp172[*]	HVH 126 (4-6034225)	GCA_000457835.1
pksp173[*]	HVH 127 (4-7303629)	GCA_000457855.1
pksp174	HVH 128 (4-7030436)	GCA_000457875.1
pksp175	HVH 132 (4-6876862)	GCA_000457915.1
pksp176[*]	HVH 137 (4-2124971)	GCA_000457995.1
pksp177	HVH 138 (4-6066704)	GCA_000458015.1

ID	Isolate	Assembly
pksp178	HVH 142 (4-5627451)	GCA_000458095.1
pksp179	HVH 143 (4-5674999)	GCA_000458115.1
pksp180	HVH 144 (4-4451937)	GCA_000458135.1
pksp181	HVH 149 (4-4451880)	GCA_000458215.1
pksp182	HVH 156 (4-3206505)	GCA_000458335.1
pksp183	HVH 157 (4-3406229)	GCA_000458355.1
pksp184	HVH 159 (4-5818141)	GCA_000458395.1
pksp185	HVH 160 (4-5695937)	GCA_000458415.1
pksp186	HVH 161 (4-3119890)	GCA_000458435.1
pksp187	HVH 169 (4-1075578)	GCA_000458535.1
pksp188	HVH 171 (4-3191958)	GCA_000458575.1
pksp189	HVH 172 (4-3248542)	GCA_000458605.1
pksp190	HVH 185 (4-2876639)	GCA_000458765.1
pksp191[*]	HVH 192 (4-3054470)	GCA_000458895.1
pksp192	HVH 197 (4-4466217)	GCA_000458995.1
pksp193[*]	HVH 199 (4-5670322)	GCA_000459035.1
pksp194	HVH 204 (4-3112802)	GCA_000459135.1

ID	Isolate	Assembly
pksp195	HVH 207 (4-3113221)	GCA_000459195.1
pksp196[*]	HVH 210 (4-3042480)	GCA_000459255.1
pksp197[*]	HVH 211 (4-3041891)	GCA_000459275.1
pksp198	HVH 212 (3-9305343)	GCA_000459295.1
pksp199	HVH 213 (4-3042928)	GCA_000459315.1
pksp200	HVH 216 (4-3042952)	GCA_000459355.1
pksp201	HVH 218 (4-4500903)	GCA_000459395.1
pksp202	HVH 220 (4-5876842)	GCA_000459415.1
pksp203	HVH 225 (4-1273116)	GCA_000459495.1
pksp204	HVH 227 (4-2277670)	GCA_000459515.1
pksp205	HVH 228 (4-7787030)	GCA_000459535.1
pksp206	KOEGE 30 (63a)	GCA_000459615.1
pksp207[*]	KOEGE 32 (66a)	GCA_000459635.1
pksp208	KOEGE 43 (105a)	GCA_000459695.1
pksp209	KOEGE 44 (106a)	GCA_000459715.1
pksp210	KOEGE 56 (169a)	GCA_000459735.1
pksp211	KOEGE 58 (171a)	GCA_000459755.1

ID	Isolate	Assembly
pksp212	KOEGE 61 (174a)	GCA_000459775.1
pksp213	KOEGE 70 (185a)	GCA_000459835.1
pksp214	UMEA 3014-1	GCA_000459955.1
pksp215	UMEA 3022-1	GCA_000459975.1
pksp216*	UMEA 3041-1	GCA_000460015.1
pksp217	UMEA 3053-1	GCA_000460055.1
pksp218	UMEA 3087-1	GCA_000460095.1
pksp219	UMEA 3088-1	GCA_000460115.1
pksp220	UMEA 3097-1	GCA_000460135.1
pksp221	UMEA 3113-1	GCA_000460175.1
pksp222	UMEA 3121-1	GCA_000460215.1
pksp223	UMEA 3122-1	GCA_000460235.1
pksp224	UMEA 3159-1	GCA_000460415.1
pksp225	UMEA 3161-1	GCA_000460455.1
pksp226	UMEA 3172-1	GCA_000460515.1
pksp227	UMEA 3173-1	GCA_000460535.1
pksp228	UMEA 3175-1	GCA_000460575.1

ID	Isolate	Assembly
pksp229	UMEA 3178-1	GCA_000460615.1
pksp230	UMEA 3185-1	GCA_000460655.1
pksp231	UMEA 3193-1	GCA_000460695.1
pksp232	UMEA 3208-1	GCA_000460815.1
pksp233	UMEA 3215-1	GCA_000460855.1
pksp234	UMEA 3216-1	GCA_000460875.1
pksp235	UMEA 3217-1	GCA_000460895.1
pksp236	UMEA 3220-1	GCA_000460915.1
pksp237	UMEA 3221-1	GCA_000460935.1
pksp238	UMEA 3222-1	GCA_000460955.1
pksp239	UMEA 3230-1	GCA_000460975.1
pksp240	UMEA 3233-1	GCA_000460995.1
pksp241	UMEA 3244-1	GCA_000461035.1
pksp242	UMEA 3257-1	GCA_000461055.1
pksp243	UMEA 3264-1	GCA_000461075.1
pksp244	UMEA 3268-1	GCA_000461095.1
pksp245*	UMEA 3298-1	GCA_000461155.1

ID	Isolate	Assembly
pksp246	UMEA 3337-1	GCA_000461275.1
pksp247	UMEA 3341-1	GCA_000461295.1
pksp248	UMEA 3391-1	GCA_000461335.1
pksp249	UMEA 3490-1	GCA_000461355.1
pksp250	UMEA 3585-1	GCA_000461375.1
pksp251	UMEA 3617-1	GCA_000461435.1
pksp252*	UMEA 3632-1	GCA_000461455.1
pksp253	UMEA 3652-1	GCA_000463605.1
pksp254	UMEA 3687-1	GCA_000461555.1
pksp255	UMEA 3694-1	GCA_000461575.1
pksp256	UMEA 3705-1	GCA_000461635.1
pksp257	UMEA 3707-1	GCA_000461655.1
pksp258	UMEA 3821-1	GCA_000461715.1
pksp259*	UMEA 3834-1	GCA_000461735.1
pksp260	UMEA 3955-1	GCA_000461815.1
pksp261	UMEA 4075-1	GCA_000461835.1
pksp262	UMEA 4076-1	GCA_000461855.1

ID	Isolate	Assembly
pksp263	UMEA 4207-1	GCA_000461875.1
pksp264	907391	GCA_000488315.1
pksp265	907892	GCA_000488475.1
pksp266*	908675	GCA_000488755.1
pksp267	910096-2	GCA_000488795.1
pksp268	A25922R	GCA_000488815.1
pksp269	A35218R	GCA_000488835.1
pksp270	UMEA 3426-1	GCA_000488075.1
pksp271	UMEA 3290-1	GCA_000488095.1
pksp272	UMEA 3693-1	GCA_000488115.1
pksp273	UMEA 3342-1	GCA_000488155.1
pksp274	LAU-EC6	GCA_000506445.2
pksp275	HVH 23 (4-6066488)	GCA_000507605.1
pksp276	HVH 83 (4-2051087)	GCA_000507625.1
pksp277	JCM 5491	GCA_000614625.1
pksp278	Nissle 1917	GCA_000333215.1
pksp279*	A192PP	GCA_001245225.1
pksp280	7996-1	GCA_000699365.1
pksp281	UCD_JA17	GCA_000599745.2
pksp282	UCD_JA23	GCA_000599765.2
pksp283	BIDMC 83	GCA_000633655.1
pksp284	2009-46	GCA_000696545.1
pksp285	UCD_JA17_pb	GCA_000714915.1

ID	Isolate	Assembly
pksp286	UCD_JA23_pb	GCA_000715035.1
pksp287*	SCB12	GCA_000817355.1
pksp288*	BIDMC 65	GCA_000692475.1
pksp289*	3-105-05_S4_C2	GCA_000700145.1
pksp290	4-203-08_S1_C1	GCA_000700705.1
pksp291	8-415-05_S4_C1	GCA_000711455.1
pksp292	8-415-05_S4_C2	GCA_000711365.1
pksp293	8-415-05_S4_C3	GCA_000711435.1
pksp294	4-203-08_S1_C2	GCA_000713945.1
pksp295	4-203-08_S1_C3	GCA_000713975.1
pksp296	8-415-05_S3_C3	GCA_000713455.1
pksp297	8-415-05_S3_C1	GCA_000713495.1
pksp298	8-415-05_S3_C2	GCA_000713585.1
pksp299	upec-98	GCA_000776315.1
pksp300	upec-93	GCA_000776695.1
pksp301	upec-91	GCA_000776855.1
pksp302	upec-9	GCA_000776795.1
pksp303	upec-87	GCA_000776745.1
pksp304	upec-85	GCA_000776455.1
pksp305	upec-84	GCA_000776215.1
pksp306	upec-80	GCA_000776035.1

ID	Isolate	Assembly
pksp307*	upec-8	GCA_000776195.1
pksp308	upec-79	GCA_000776415.1
pksp309	upec-77	GCA_000776155.1
pksp310*	upec-76	GCA_000776235.1
pksp311*	upec-75	GCA_000776655.1
pksp312*	upec-73	GCA_000776375.1
pksp313	upec-7	GCA_000776175.1
pksp314	upec-65	GCA_000776615.1
pksp315*	upec-61	GCA_000776565.1
pksp316	upec-60	GCA_000776505.1
pksp317*	upec-51	GCA_000776965.1
pksp318	upec-48	GCA_000777025.1
pksp319	upec-40	GCA_000777135.1
pksp320	upec-39	GCA_000777165.1
pksp321	upec-38	GCA_000777195.1
pksp322	upec-36	GCA_000777215.1
pksp323	upec-289	GCA_000777415.1
pksp324	upec-288	GCA_000777435.1
pksp325	upec-287	GCA_000777455.1
pksp326	upec-285	GCA_000777495.1
pksp327	upec-277	GCA_000777605.1
pksp328	upec-276	GCA_000777625.1
pksp329	upec-261	GCA_000777845.1
pksp330	upec-260	GCA_000777895.1
pksp331	upec-258	GCA_000777975.1
pksp332*	upec-255	GCA_000778035.1
pksp333	upec-253	GCA_000778075.1
pksp334	upec-251	GCA_000778095.1

ID	Isolate	Assembly
pksp335*	upec-250	GCA_000778105.1
pksp336*	upec-249	GCA_000778135.1
pksp337	upec-244	GCA_000778215.1
pksp338	upec-237	GCA_000778335.1
pksp339	upec-236	GCA_000778355.1
pksp340	upec-232	GCA_000778415.1
pksp341	upec-230	GCA_000778435.1
pksp342	upec-229	GCA_000776715.1
pksp343	upec-228	GCA_000778815.1
pksp344	upec-226	GCA_000778685.1
pksp345	upec-225	GCA_000778955.1
pksp346*	upec-209	GCA_000778465.1
pksp347	upec-201	GCA_000779255.1
pksp348*	upec-197	GCA_000779425.1
pksp349	upec-193	GCA_000779545.1
pksp350	upec-186	GCA_000779585.1
pksp351	upec-184	GCA_000779715.1
pksp352	upec-181	GCA_000779795.1
pksp353	upec-172	GCA_000779995.1
pksp354*	upec-169	GCA_000780095.1
pksp355	upec-166	GCA_000780115.1
pksp356	upec-161	GCA_000780155.1
pksp357	upec-158	GCA_000780195.1
pksp358*	upec-157	GCA_000780215.1
pksp359	upec-156	GCA_000780235.1
pksp360	upec-153	GCA_000780335.1
pksp361*	upec-144	GCA_000780595.1
pksp362	upec-140	GCA_000780735.1

ID	Isolate	Assembly
pksp363	upec-14	GCA_000780675.1
pksp364*	upec-139	GCA_000780755.1
pksp365	upec-138	GCA_000780775.1
pksp366*	upec-136	GCA_000780695.1
pksp367	upec-135	GCA_000780795.1
pksp368*	upec-131	GCA_000780875.1
pksp369*	upec-129	GCA_000780925.1
pksp370*	upec-124	GCA_000781035.1
pksp371	upec-123	GCA_000781045.1
pksp372*	upec-120	GCA_000781095.1
pksp373	upec-117	GCA_000781175.1
pksp374	upec-115	GCA_000781215.1
pksp375	upec-109	GCA_000781355.1
pksp376*	upec-106	GCA_000781385.1
pksp377	upec-10	GCA_000785355.1
pksp378	blood-11-0041	GCA_000779495.1
pksp379*	blood-11-0031	GCA_000780275.1
pksp380	blood-10-1386	GCA_000779615.1
pksp381	blood-10-1310	GCA_000778765.1
pksp382	blood-10-1308	GCA_000778915.1
pksp383	blood-10-1126	GCA_000779125.1
pksp384	blood-10-1105	GCA_000779025.1
pksp385*	blood-10-0687	GCA_000781555.1

ID	Isolate	Assembly
pksp386*	blood-10-0686	GCA_000781575.1
pksp387*	blood-09-0751	GCA_000782055.1
pksp388	blood-08-1203	GCA_000782635.1
pksp389	blood-08-0997	GCA_000782655.1
pksp390*	blood-08-0654	GCA_000782695.1
pksp391*	blood-08-0493	GCA_000782735.1
pksp392	blood-08-0379	GCA_000782755.1
pksp393	blood-08-0215	GCA_000782775.1
pksp394	UPEC_011	GCA_001651725.1
pksp395	UPEC_008	GCA_001651625.1
pksp396	UPEC_001	GCA_001651715.1
pksp397	GSK25213	GCA_000807565.1
pksp398	GSK2528	GCA_000807635.1
pksp399	SCB11	GCA_000817375.1
pksp400	GSK2522	GCA_000807575.1
pksp401	GSK2524	GCA_000807555.1
pksp402	GSK252FU	GCA_000807655.1
pksp403	GSK252BU	GCA_000800675.1
pksp404	932_ECOL	GCA_001059575.1
pksp405	696_ECOL	GCA_001057995.1
pksp406	502_ECOL	GCA_001057065.1
pksp407*	417_ECOL	GCA_001056665.1
pksp408*	121_ECOL	GCA_001054095.1

ID	Isolate	Assembly
pksp409	1187_ECOL	GCA_001076105.1
pksp410	11_ECOL	GCA_001052125.1
pksp411*	RS218	GCA_000817345.1
pksp412	VACI-14	GCA_001448025.1
pksp413	M17 - 1	GCA_001010195.1
pksp414*	LSPQ A134697	GCA_001262455.1
pksp415	BWH59	GCA_001030285.1
pksp416	MGH122	GCA_001030435.1
pksp417	BIDMC97	GCA_001030445.1
pksp418*	BIDMC114	GCA_001030665.1
pksp419	UCD-JA09	GCA_001306575.1
pksp420	UCD-JA19	GCA_001306585.1
pksp421	UCD-JA30	GCA_001306685.1
pksp422*	UCD-JA38	GCA_001306635.1
pksp423	50639799	GCA_001463205.1
pksp424	50870281	GCA_001463455.1
pksp425	STEC 1528	GCA_001608125.1
pksp426*	GN02005	GCA_001519135.1
pksp427	GN02007	GCA_001519115.1
pksp428	GN02009	GCA_001519125.1
pksp429	GN02045	GCA_001519215.1
pksp430*	GN02099	GCA_001519715.1
pksp431	GN02137	GCA_001519675.1
pksp432*	GN02148	GCA_001519755.1
pksp433	GN02163	GCA_001519475.1
pksp434*	GN02165	GCA_001519285.1
pksp435*	GN02172	GCA_001519235.1

ID	Isolate	Assembly
pksp436	GN02183	GCA_001519315.1
pksp437*	GN02254	GCA_001519735.1
pksp438*	GN02260	GCA_001519555.1
pksp439	GN02289	GCA_001521215.1
pksp440	GN02314	GCA_001521195.1
pksp441	GN02323	GCA_001519595.1
pksp442	GN02350	GCA_001521225.1
pksp443	GN02370	GCA_001520015.1
pksp444	GN02392	GCA_001520055.1
pksp445	GN02411	GCA_001520895.1
pksp446	GN02529	GCA_001520215.1
pksp447	GN02547	GCA_001521355.1
pksp448*	GN02627	GCA_001520195.1
pksp449	GN02639	GCA_001519645.1
pksp450	GN02766	GCA_001520815.1
pksp451	GN02787	GCA_001521155.1
pksp452	GN02867	GCA_001521015.1
pksp453*	GN03324	GCA_001521575.1
pksp454	GN03398	GCA_001519485.1
pksp455*	GN03409	GCA_001520775.1
pksp456	GN03545	GCA_001520715.1
pksp457	GN03661	GCA_001521115.1
pksp458	GN03786	GCA_001521315.1
pksp459*	GN04262	GCA_001521455.1
pksp460	GN02748	GCA_001518355.1
pksp461*	GN02487	GCA_001524905.1
pksp462	UM149	GCA_001571585.1
pksp463	UM131	GCA_001571575.1

ID	Isolate	Assembly
pksp464*	UM141	GCA_001571565.1
pksp465	UC37	GCA_001571745.1
pksp466	JPH264	GCA_001562835.1
pksp467	sheep1	GCA_001615225.1
pksp468	sheep6	GCA_001614495.1
pksp469	sheep17	GCA_001616475.1
pksp470	GN04499	GCA_001620985.1
pksp471	GN04772	GCA_001621225.1
pksp472	GN05109	GCA_001621345.1
pksp473	GN05681	GCA_001621675.1
pksp474	GN05963	GCA_001621885.1
pksp475	GN05992	GCA_001621915.1
pksp476	GN06113	GCA_001621995.1
pksp477*	GN06168	GCA_001622105.1
pksp478	NGF2	GCA_001683595.1
pksp479	NGF3	GCA_001683585.1
pksp480	NGF4	GCA_001683575.1
pksp481	1.41E+09	GCA_001692775.1
pksp482	1.41E+09	GCA_001692865.1
pksp483	1.51E+09	GCA_001692805.1
pksp484	1.51E+09	GCA_001692785.1
pksp485	Fec 67	GCA_001865185.1
pksp486*	SF-384	GCA_001877815.1
pksp487*	SF-452	GCA_001877805.1
pksp488	No.12	GCA_001865915.1
pksp489	80//6	GCA_001865925.1
pksp490	B-11870	GCA_001865985.1
pksp491*	SF-491	GCA_001881225.1

ID	Isolate	Assembly
pksp492	SF-495	GCA_001881235.1
pksp493*	SF-518	GCA_001881245.1
pksp494*	SF-522	GCA_001881055.1
pksp495	SF-523	GCA_001881275.1
pksp496*	SF-560	GCA_001881305.1
pksp497*	SF-567	GCA_001881315.1
pksp498*	SF-572	GCA_001881355.1
pksp499*	SF-596	GCA_001881345.1
pksp500*	SF-626	GCA_001881075.1
pksp501*	SF-095	GCA_001881385.1
pksp502*	SF-126	GCA_001881105.1
pksp503*	MVAST0098	GCA_001881125.1
pksp504*	MVAST0176	GCA_001881395.1
pksp505	MVAST0234	GCA_001881425.1
pksp506*	USVAST184	GCA_001881155.1
pksp507*	USVAST245	GCA_001881435.1
pksp508*	USVAST267	GCA_001881165.1
pksp509*	USVAST356	GCA_001881465.1
pksp510*	USVAST406	GCA_001881205.1
pksp511*	F-18	GCA_001854565.1
pksp512	CFT073	GCA_000007445.1
pksp513*	UTI89	GCA_000013265.1
pksp514	536	GCA_000013305.1
pksp515*	IHE3034	GCA_000025745.1
pksp516	ABU 83972	GCA_000148365.1
pksp517	UM146	GCA_000148605.1
pksp518	clone D i2	GCA_000233875.1
pksp519	clone D i14	GCA_000233895.1

ID	Isolate	Assembly
pksp520	PMV-1	GCA_000493595.1
pksp521	Nissle 1917	GCA_000714595.1
pksp522	ATCC 25922	GCA_000743255.1
pksp523	RS218	GCA_000800845.2
pksp524*	SF-166	GCA_001280385.1
pksp525*	SF-173	GCA_001280405.1
pksp526	NGF1	GCA_001660585.1
pksp527	ECONIH2	GCA_001675145.1
pksp528	K-15KW01	GCA_001683435.1
pksp529	UPEC 26-1	GCA_001693315.1
pksp530	D8	GCA_001900395.1
