# Supplementary material for: Evolutionary Dynamics Based on Comparative Genomics of Pathogenic Escherichia coli Lineages Harboring Polyketide Synthase (pks) Island
Source: mBio. 2021 Mar 2;12(1):e03634-20. doi: 10.1128/mBio.03634-20 (PMC8545132; doi:10.1128/mBio.03634-20)
Supplement: TABLE S6 [file mbio.03634-20-st006.pdf]

Table S6: Isolate names and accession IDs of in-house *pks* negative genomes used *in silico* virulence/resistance gene profiling (Sl. No.s 1 to 23) and ST95 *pks* negative genomes from NCBI (Sl. No.s 24 to 72)

Sl. No.	ID	Isolate	Assembly
1.	NA1004	NA1004	GCA_002918085.1
2.	NA023	NA023	GCA_002224755.1
3.	NA057	NA057	GCA_002224705.1
4.	NA081	NA081	GCA_002224715.1
5.	NA101	NA101	GCA_002224795.1
6.	NA112	NA112	GCA_002224745.1
7.	NA447	NA447	GCA_002224785.1
8.	NA084	NA084	GCA_001713585.1
9.	NA086	NA086	GCA_001713575.1
10.	NA099	NA099	GCA_001713555.1
11.	NA703	NA703	GCA_001713545.1
12.	NA724	NA724	GCA_001713625.1
13.	NA114	NA114	GCA_000214765.3
14.	NA1001	NA1001	GCA_002918015.1
15.	NA1002	NA1002	GCA_002918005.1
16.	NA1003	NA1003	GCA_002918075.1
17.	NAEC6	NAEC6	GCA_002918095.1
18.	NAEC1	NAEC1	GCA_002918065.1
19.	NAEC2	NAEC2	GCA_002918175.1
20.	NAEC3	NAEC3	GCA_002918145.1
21.	NAEC4	NAEC4	GCA_002918155.1
22.	NAEC5	NAEC5	GCA_002918165.1
23.	NA090	NA090	GCA_002407385.1
24.	95N001	H252	GCA_000190895.1
25.	95N002	DSM 30083	GCA_000690815.1
26.	95N003	KTE4	GCA_000350645.1
27.	95N004	KTE5	GCA_000350665.1
28.	95N005	KTE62	GCA_000351605.1
29.	95N006	KTE3	GCA_000407685.1
30.	95N007	KTE7	GCA_000407705.1
31.	95N008	KTE27	GCA_000407885.1
32.	95N009	KTE240	GCA_000408305.1
33.	95N010	HVH 5 (4-7148410)	GCA_000456085.1
34.	95N011	HVH 32 (4-3773988)	GCA_000456505.1
35.	95N012	HVH 59 (4-1119338)	GCA_000456885.1
36.	95N013	HVH 73 (4-2393174)	GCA_000457025.1
37.	95N014	HVH 102 (4-6906788)	GCA_000465155.1
38.	95N015	HVH 104 (4-6977960)	GCA_000457455.1
39.	95N016	HVH 148 (4-3192490)	GCA_000495015.1
40.	95N017	HVH 170 (4-3026949)	GCA_000458555.1
41.	95N018	HVH 178 (4-3189163)	GCA_000495055.1
42.	95N019	HVH 180 (4-3051617)	GCA_000458685.1
43.	95N020	HVH 191 (3-9341900)	GCA_000458875.1
44.	95N021	HVH 201 (4-4459431)	GCA_000459075.1
45.	95N022	HVH 203 (4-3126218)	GCA_000459115.1

Sl. No.	ID	Isolate	Assembly
46.	95N023	HVH 217 (4-1022806)	GCA_000459375.1
47.	95N024	HVH 222 (4-2977443)	GCA_000459455.1
48.	95N025	UMEA 3140-1	GCA_000460295.1
49.	95N026	UMEA 3203-1	GCA_000460775.1
50.	95N027	UMEA 3206-1	GCA_000460795.1
51.	95N028	UMEA 3662-1	GCA_000461495.1
52.	95N029	UMEA 3702-1	GCA_000461595.1
53.	95N030	UMEA 3893-1	GCA_000461775.1
54.	95N031	597	GCA_000503475.1
55.	95N032	HVH 214 (4-3062198)	GCA_000507665.1
56.	95N033	AL505	GCA_001499595.1
57.	95N038	BIDMC 49b	GCA_000522365.1
58.	95N039	BIDMC 49a	GCA_000522385.1
59.	95N040	ATCC 11775	GCA_000734955.1
60.	95N041	upec-185	GCA_000779695.1
61.	95N042	50857972	GCA_001463405.1
62.	95N043	GN02476	GCA_001520875.1
63.	95N044	GN04665	GCA_001621085.1
64.	95N045	GN04676	GCA_001621125.1
65.	95N046	GN05696	GCA_001621665.1
66.	95N047	018PP2015	GCA_001700095.1
67.	95N048	SF-501	GCA_001881045.1
68.	95N049	MVAST0326	GCA_001881145.1
69.	95N034	SF-088	GCA_001280325.1
70.	95N035	SF-468	GCA_001280345.1
71.	95N036	APEC O1	GCA_000014845.1
72.	95N037	S88	GCA_000026285.1
