# Supplementary material for: Evolutionary Dynamics Based on Comparative Genomics of Pathogenic Escherichia coli Lineages Harboring Polyketide Synthase (pks) Island
Source: mBio. 2021 Mar 2;12(1):e03634-20. doi: 10.1128/mBio.03634-20 (PMC8545132; doi:10.1128/mBio.03634-20)
Supplement: TABLE S7 [file mbio.03634-20-st007.pdf]

Table S7: Table showing the COG classification of core genes from 159 ST95 genomes

COG CLASSIFICATION	NUMBER OF GENES
**CELLULAR PROCESSES AND SIGNALING**	
**[D] Cell cycle control, cell division, chromosome partitioning**	**32**
**[M] Cell wall/membrane/envelope biogenesis**	**177**
**[N] Cell motility**	**22**
**[O] Post-translational modification, protein turnover, and chaperones**	**119**
**[T] Signal transduction mechanisms**	**89**
**[U] Intracellular trafficking, secretion, and vesicular transport**	**52**
**[V] Defence mechanisms**	**40**
**INFORMATION STORAGE AND PROCESSING**	
**[A] RNA processing and modification**	**2**
**[J] Translation, ribosomal structure and biogenesis**	**153**
**[K] Transcription**	**232**
**[L] Replication, recombination and repair**	**102**
**METABOLISM**	
**[C] Energy production and conversion**	**228**
**[E] Amino acid transport and metabolism**	**219**
**[F] Nucleotide transport and metabolism**	**103**
**[G] Carbohydrate transport and metabolism**	**280**
**[H] Coenzyme transport and metabolism**	**124**
**[I] Lipid transport and metabolism**	**71**
**[P] Inorganic ion transport and metabolism**	**216**
**[Q] Secondary metabolites biosynthesis, transport, and catabolism**	**24**
**MULTIPLE CLASSES**	**52**
**POORLY CHARACTERIZED**	
**[S] Function unknown**	**649**
**Unidentified**	**71**
